# Inhibitory Effect of Synthetic Flavone Derivatives on Pan-Aurora Kinases: Induction of G2/M Cell-Cycle Arrest and Apoptosis in HCT116 Human Colon Cancer Cells

**DOI:** 10.3390/ijms19124086

**Published:** 2018-12-17

**Authors:** Soon Young Shin, Youngshim Lee, Beom Soo Kim, Junho Lee, Seunghyun Ahn, Dongsoo Koh, Yoongho Lim, Young Han Lee

**Affiliations:** 1 Department of Biological Sciences, Sanghuh College of Life Science s, Konkuk University, Seoul 05029, Korea; shinsy@konkuk.ac.kr; 2 Cancer and Metabolism Institute, Konkuk University, Seoul 05029, Korea; 3 Division of Bioscience and Biotechnology, BMIC, Konkuk University, Seoul 05029, Korea; librashim@gmail.com (Y.L.); kimbs0812@naver.com (B.S.K.); cttyofjoy@naver.com (J.L.); mistahn321@naver.com (S.A.); yoongho@konkuk.ac.kr (Y.L.); 4 Department of Applied Chemistry, Dongduk Women’s University, Seoul 02748, Korea; dskoh@dongduk.ac.kr

**Keywords:** flavones, colon cancer, clonogenicity, apoptosis, aurora kinases, quantitative structure–activity relationship, in-silico docking

## Abstract

Members of the aurora kinase family are Ser/Thr kinases involved in regulating mitosis. Multiple promising clinical trials to target aurora kinases are in development. To discover flavones showing growth inhibitory effects on cancer cells, 36 flavone derivatives were prepared, and their cytotoxicity was measured using a long-term clonogenic survival assay. Their half-maximal growth inhibitory effects against HCT116 human colon cancer cells were observed at the sub-micromolar level. Pharmacophores were derived based on three-dimensional quantitative structure–activity calculations. Because plant-derived flavones inhibit aurora kinase B, we selected 5-methoxy-2-(2-methoxynaphthalen-1-yl)-4*H*-chromen-4-one (derivative **31**), which showed the best half-maximal cell growth inhibitory effect, and tested whether it can inhibit aurora kinases in HCT116 colon cancer cells. We found that derivative **31** inhibited the phosphorylation of aurora kinases A, aurora kinases B and aurora kinases C, suggesting that derivative **31** is a potential pan-aurora kinase inhibitor. The results of our analysis of the binding modes between derivative **31** and aurora A and aurora B kinases using in-silico docking were consistent with the pharmacophores proposed in this study.

## 1. Introduction

Flavones are a class of flavonoids having a backbone of 2-phenylchromen-4-one (2-phenyl-1-benzopyran-4-one). They are common in fruits and many plant foods. Some natural flavones show anticancer activity. For example, apigenin (4′,5,7-trihydroxyflavone), luteolin (3′,4′,5,7-tetrahydroxyflavone), baicalein (5,6,7-trihydroxyflavone), nobiletin (3′,4′,5,6,7,8-hexamethoxyflavone) and tangeretin (4′,5,6,7,8-pentamethoxyflavone) inhibit the proliferation of breast cancer cells [1,2,3]. Kaempferol (3,4′,5,7-tetrahydroxyflavone) suppresses the growth of bladder and cervical cancer cells [4]. Chrysin (5,7-dihydroxyflavone) reduces the proliferation of prostate cancer cells [5]. Quercetagetin (3,3′,4′,5,6,7-hexahydroxyflavone) induces apoptosis in colon cancer cells [6]. However, the structural features of flavones exhibiting inhibitory effects on cancer cell growth remain unclear.

In order to identify the structural characteristics of flavones exhibiting growth inhibitory effects on cancer cells, we prepared 36 synthetic flavone derivatives containing hydroxy, fluoro, bromo, nitro, methoxy, methyl, styryl or naphthalenyl groups [7]. Colon cancer is the second most diagnosed cancer in women and the third in men [8]. It is also the second most death-causing cancer [8]. Tumor penetration and metastasis can occur in stage II of colon cancer; thus, its survival rate is approximately 70% after stage II. Even when the resection of colon cancer was achieved by the first treatment of colon cancer owing to diagnosis at the early stage, chemotherapy is often required simultaneously with other treatments [9]. Several drugs have been approved as colon cancer chemotherapy. Drugs as adjuvant chemotherapy after surgery are being developed [10]. Here, we used a long-term clonogenic assay to measure the growth inhibitory effects of synthetic flavones in HCT116 human colon cancer cells. The half-maximal cell growth inhibitory concentration (GI_50_) values of 36 flavone derivatives against HCT116 cells were determined. To derive structural features that exhibit better cell growth inhibitory effects, relationships between the structural properties of the synthetic flavones and their cell growth inhibitory effects were calculated using comparative molecular field analysis and comparative molecular similarity index analysis. Among the 36 synthetic flavone derivatives, we found that 5-methoxy-2-(2-methoxynaphthalen-1-yl)-4*H*-chromen-4-one (named derivative **31**) showed the best GI_50_ value (0.49 μM). Because we have shown in our previous study that plant-derived flavones inhibit aurora kinase B (AURKB) [6], we further evaluated the effect of derivative **31** on the inhibition of aurora kinases. The molecular binding modes between the derivative **31** compound and aurora kinases were elucidated using in-silico docking experiments. These results can provide valuable information for designing novel anticancer drugs that target aurora kinases.

## 2. Results and Discussion

To investigate the structural features of flavones that influence the growth of cancer cells, we synthesized 36 flavone derivatives and screened candidate compounds by monitoring their growth inhibitory efficacy by a long-term clonogenic survival assay [11], which can efficiently distinguish differences in cell growth rate induced by structurally similar compounds [12,13].

**Figure 1:**
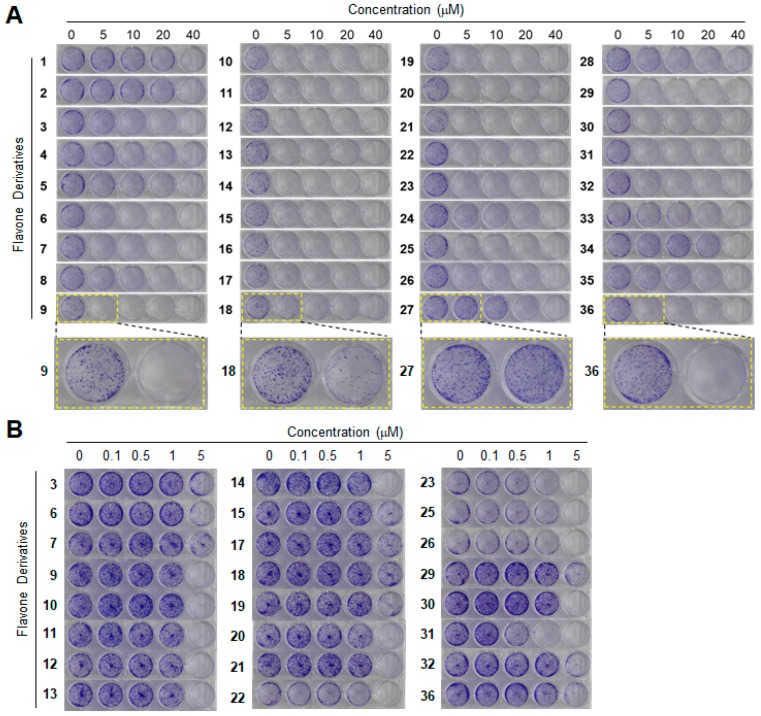
Effects of flavone derivatives on the inhibition of clonogenicity of HCT116 colon cancer cells. Cells were treated with derivative compounds at 0, 5, 10, 20 and 40 μM (**A**) or at 0, 0.1, 0.5, 1 and 5 μM (**B**). The dashed lines show the enlarged images.

**Figure 2:**
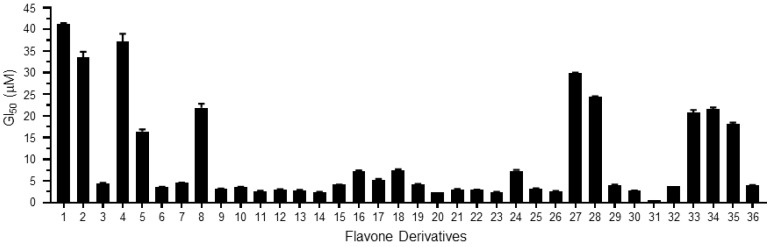
Half-maximal cell growth inhibitory concentration (GI_50_) values in Table 1.

Initially, we tested the inhibitory activity of the derivatives at high (0, 5, 10, 20 and 40 μM) (Figure 1A) and low concentrations (0, 0.1, 0.5, 1 and 5 μM) (Figure 1B) on the clonogenicity of cancer cells. Clonogenicity was quantitated by densitometry, GI_50_ values were computed using the SigmaPlot software, and the results are shown in Table 1 and Figure 2.

Their GI_50_ values ranged between 0.49 and 41.19 μM. Among the derivatives, derivative **31**, 5-methoxy-2-(2-methoxynaphthalen-1-yl)-4*H*-chromen-4-one, showed the most effective inhibition of clonogenicity (GI_50_ value: 0.49 μM). Negative logarithmic scales of the GI_50_ values (pGI_50_) were used as biological data for 3D-QSAR calculation. The 3D structures of the derivatives required for 3D-QSAR calculations were modified using the Sybyl 7.3 program from the X-ray crystallographic structures of derivatives **6** (2-(3,4-dimethoxyphenyl)-3-hydroxy-4*H*-chromen-4-one) and **18** (2-(2,3-dimethoxynaphthalen-1-yl)-3-hydroxy-6-methoxy-4*H*-chromen-4-one), which were determined by the authors’ previous works [14,15]. Three-dimensional quantitative structure–activity relationship (3D-QSAR) was performed using comparative molecular field analysis (CoMFA) and comparative molecular similarity indices analysis (CoMSIA). We divided the 36 derivatives into a training set and a test set. The former was used to generate QSAR models and the latter was used to validate the models generated by the training set. Seven derivatives, namely **7**, **10**, **14**, **17**, **22**, **29** and **32,** were arbitrarily chosen for the test set by one of the data analysis tools, hierarchical clustering analysis [16], as shown in Figure S1. Twenty-nine derivatives in the training set were aligned using the Sybyl DATABASE Alignment module. They were aligned well, which indicated interactions between probe atoms and rest atoms (Figure S2). Linear correlations between structural properties of the derivatives in the training set and their cancer cell growth inhibitory effects were determined using partial least-squares regression. Among the many CoMFA models generated by iteration, until a good cross-validation correlation coefficient (q^2^) was found, the model showing 0.772 of q^2^ was chosen, where non-cross-validated correlation coefficient (r^2^), optimal number of components, standard error of estimate, and F value were 0.980, 6, 0.078, and 175.833, respectively. pGI_50_ values were predicted based on this model. A comparison of the pGI_50_ values obtained from the long-term clonogenic survival assay with the values predicted using the CoMFA model is listed in Table S1, and its graph is shown in Figure S3. The residuals between the experimental data and the predicted values ranged from 0.11% to 8.29%. Likewise, the pGI_50_ values of the derivatives contained in the test set were calculated using the same CoMFA model (Table S1). The residuals between the experimental data and the predicted values ranged from 6.79% to 24.96%. These results showed that this CoMFA model was reliable. In this model, the steric and electrostatic field descriptors were 51.7% and 48.3%, respectively. To visualize the field descriptors, we generated contour maps. The steric-bulk-favoring and -disfavoring regions occupied 92% and 8% of the maps, respectively (Figure S4A). In the steric field map of the CoMFA model, green contours were shown at C2′ and C3′ positions, meaning that a bulky group was favored in the region. Derivatives containing bulky naphthyl group at the region, such as derivatives **15**–**18**, **22**–**26**, **29**–**32** and **36,** showed good GI_50_ values, which were 7.51 µM and less. Derivatives **9**–**14** have the bulky resveratrol group at C2′ and showed good activities, with GI_50_ values ranging between 3.63 µM and 2.41 µM. On the contrary, a yellow contour was observed near C4′, meaning that a bulky group was not favored in the region. It is explicable why some of the derivatives containing methoxy group at the C4′ position, such as derivatives **4**, **8**, **33**, **34** and **35**, showed poor GI_50_ values. The electrostatic contour map of the CoMFA model is shown in the Figure S4B. The electrostaticly favoring and disfavoring regions occupied 1% and 99% of the maps, respectively. In the electrostatic field map of the CoMFA model, a small red contour was observed around C2′. This may be the reason derivatives with fluoride or methoxy substitutes at C2′ showed low biological activities, as shown by derivatives **1**, **2**, **5** and **8**.

**Figure 3:**
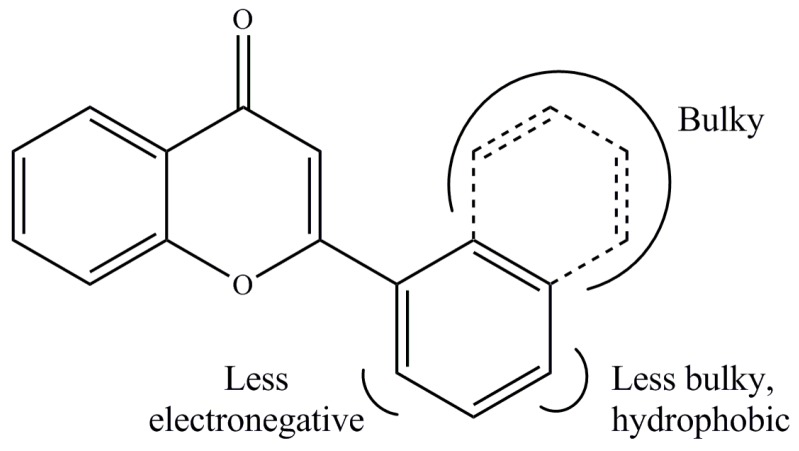
Pharmacophores derived based on the CoMFA and CoMSIA models.

Unlike CoMFA, CoMSIA provides more field descriptors, including hydrogen bond donor and acceptor descriptors and hydrophobic descriptors. To find the best CoMSIA model, many CoMSIA models were generated by iteration. The CoMSIA model showing q^2^ of 0.515 was selected, where the r^2^, optimal number of components, standard error of estimate, and F value were 0.952, 6, 0.119, and 73.084, respectively. This model consisted of steric, electrostatic and hydrophobic descriptors, and their contributions were 18.3%, 53.3% and 28.4%, respectively. The pGI_50_ values calculated using this model were compared with those obtained from the experimental values (Table S2), and they were graphed as shown in Figure S5. The residuals between the experimental data and predicted values ranged from 0.18% to 10.62%. Likewise, the pGI_50_ values of derivatives contained in the test set were calculated using the same CoMFA model (Table S2). The residuals between the experimental data and predicted values ranged from 7.34% to 23.92%. These results showed that this CoMSIA model was reliable. The steric and electrostatic field descriptors were generated to be similar to those obtained from the CoMFA model. A hydrophobic field map (Figure S6) was additionally obtained from the CoMSIA model. The hydrophobic region occupied 95% of the contour map area, whereas the nonhydrophobic region occupied 5%. The orange-colored contour around C4′ suggested that a hydrophobic group was favored at the position. The derivatives **33**–**35** had a methoxy substituent at the C4′ of thier naphthyl moiety and showed lower activities compared to derivatives **22**–**26**, which had naphthyl without a hydrophilic substituent. The pharmacophores obtained based on the CoMFA and CoMSIA models are summarized in Figure 3, which provides insights for rational design of compounds with good inhibitory effects on cell growth. In summary, a bulky group was favored at C2′ and C3′ but was not favored at C4′. A hydrophobic group was favored at C4′, and an electronegative group was not favored at C2′. One of the criteria for the solubility is logP. The logP values of derivatives containing a 3-hydroxy group ranged between 1.53 and 3.02, those of derivatives with a styryl group, 3.72 and 5.45, and logP values of naphthoflavones ranged from 3.69 to 4.07.

**Figure 4:**
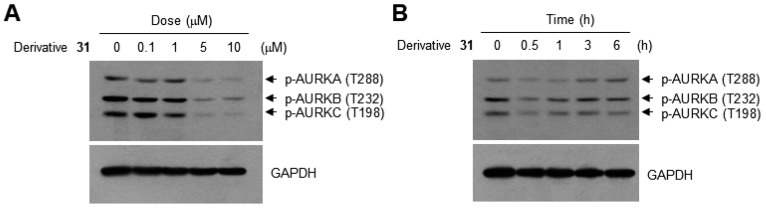
Effect of derivative **31** on inhibition of aurora kinases. HCT116 cells were serum-starved for 24 h in media containing 0.5% FBS, and treated with different concentrations of derivative **31** (0, 0.1, 1, 5 or 10 μM) for 3 h (**A**) or 5 μM derivative **31** for different times (1. 0.5, 1, 3 or 6 h) (**B**). Total cell lysates were immunoblotted with phospho-specific antibodies against AURKA (T288), AURKB (T232) and AURKC (T198). Anti-GAPDH antibody was used as an internal control.

Aurora kinases are Ser/Thr kinases that function as key regulators of chromosome alignment and segregation during mitosis [17]. There are three classes of aurora kinases: aurora kinase A (AURKA), aurora kinase B (AURKB) and aurora kinase C (AURKC). Previously, we showed that plant-derived flavones inhibit AURKB [6]. To investigate whether synthetic flavone derivatives inhibit aurora kinase activity, we selected one of the compounds, derivative **31**, which exhibited the best GI_50_ value, and examined its inhibitory activity against aurora kinases. aurora kinase activity was assessed by its phosphorylation status, as reported previously [18]. Treatment with derivative **31** decreased the phosphorylation of AURKA on Thr-288, AURKB on Thr-232, and AURKC on Thr-198 in a dose- (Figure 4A) and time-dependent (Figure 4B) manner, suggesting that derivative **31** exhibited pan-aurora kinase inhibitory activity.

**Figure 5:**
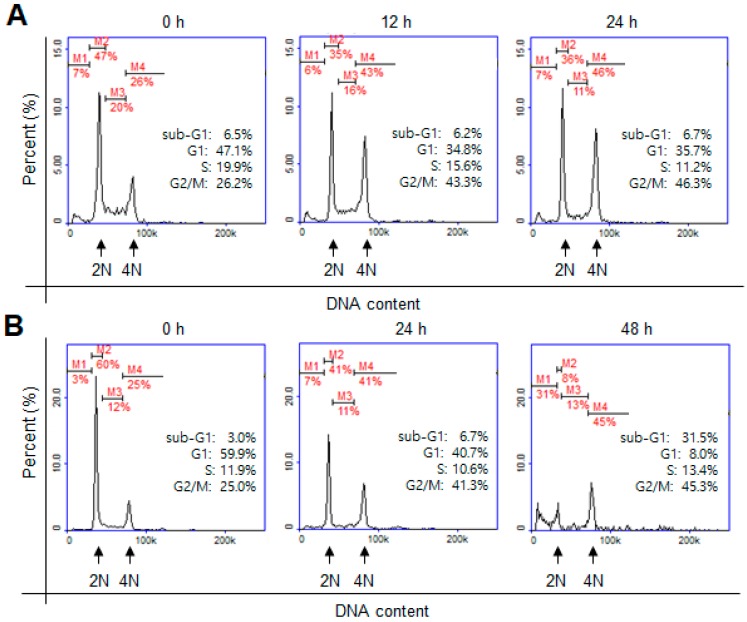
Effect of derivative **31** on G2/M arrest and apoptosis. HCT116 cells were treated with 5 μM derivative **31** for 0, 12 and 24 h (**A**) or 0, 24 and 48 h (**B**). The cells were fixed with ethanol and stained with propidium iodide (PI). Cellular DNA contents were determined by flow cytometry. 2N, diploid; 4N, tetraploid; M1, sub-G1; M2, G1; M2, S, M4, G2/M.

AURKA and AURKB are overexpressed in colon cancer [19], and inhibition of aurora kinases triggers mitotic cell-cycle arrest and apoptotic cell death [18]. Therefore, we investigated by flow cytometry whether derivative **31** affects cell cycle progression. After treatment with derivative **31,** population of G2/M phase cells increased from 26.2% (0 h) to 43.3% (12 h) and 46.3% (24 h) (Figure 5A). Notably, the number of sub-G1 phase cells remarkably increased from 3% (0 h) to 31.5% (48 h) as the number of G1 phase cells concomitantly decreased from 59.9% (0 h) to 8.0% (48 h) (Figure 5B). Because the presence of sub-G1 cell population is indicative of the progression of apoptotic cells, we suggest that derivative **31** induced cell-cycle arrest at the G2/M phase at the early stage but triggered apoptotic cell death in HCT116 colon cancer cells after continued exposure. We thus evaluated the capability of derivative **31** to induce apoptosis.

**Figure 6:**
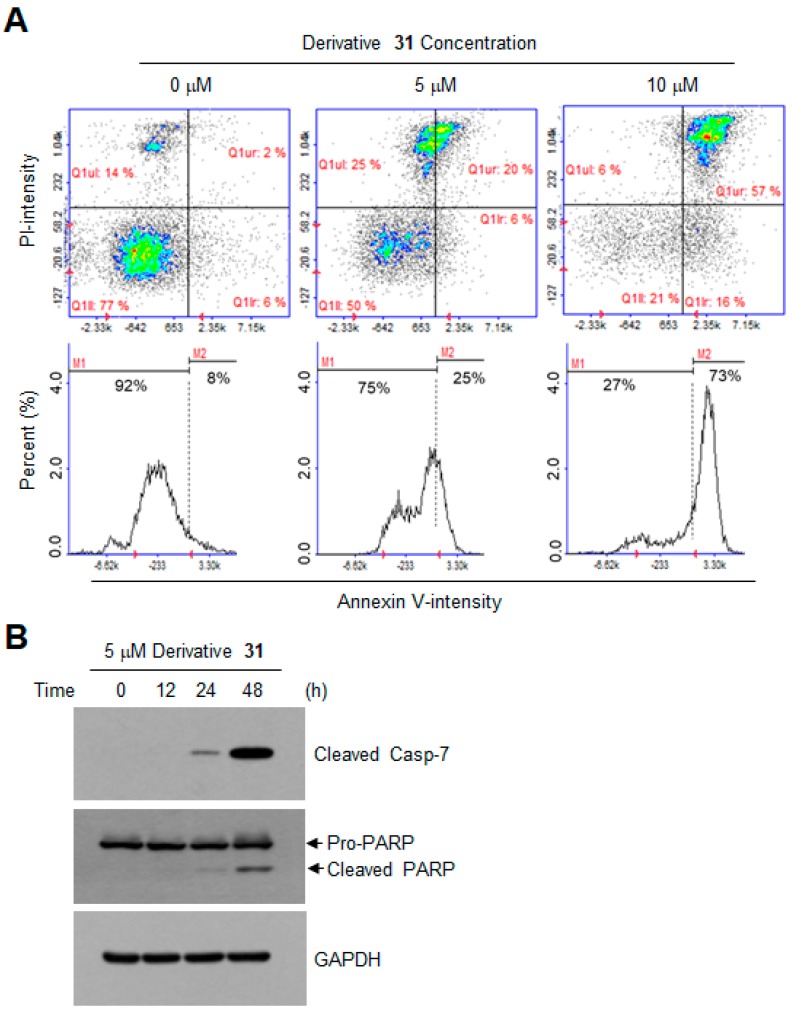
Effect of derivative **31** on apoptosis induction. (**A**) HCT116 cells were treated with derivative **31** at 0, 5 and 10 μM for 48 h, and co-stained with fluorescein isothiocyanate (FITC)–annexin V and PI. Fluorescence intensity was analyzed by a NucleoCounter NC-3000 image cytometer. Scatter plots represent FITC–annexin V versus PI intensities (upper panels). Lower graphs represent populations of annexin V-positive cells. M1, annexin V-negative; M2, annexin V-positive. (**B**) HCT116 cells were treated with 5 μM derivative **31** for 0, 6, 12 and 48 h, and total cell lysates were immunoblotted with antibodies against cleaved-caspase-7 and poly(ADP-ribose) polymerase (PARP). Anti-GAPDH antibody was used as an internal control.

Because the phosphatidylserine localized in the inner surface of the cell membrane translocates to the outer membrane during apoptosis [20], we analyzed the population of apoptotic cells by staining the outer layer of the cell membrane with phosphatidylserine by using annexin V [21]. Propidium iodide was used as a counterstain to label dead cells. Flow cytometry results showed that treatment with derivative **31** at 5 and 10 μM increased the population of annexin V-positive cells from 8% to 25% and 73%, respectively (Figure 6A). These data suggested that derivative **31** caused apoptotic cell death in HCT116 cells. Caspases regulate the cleavage of many cellular proteins, including the DNA repair enzyme poly(ADP-ribose) polymerase (PARP), to induce apoptosis [22]. Caspases are activated by proteolytic cleavages [23]. To determine whether derivative **31**-induced apoptosis is mediated by caspases, we examined the status of caspase 7 cleavage by Western blotting analysis. We found that the cleavages of caspase 7 and its substrate, PARP, were increased by treatment with derivative **31** in a time-dependent manner (Figure 6B). Taken together, derivative **31** triggered apoptosis by inhibiting aurora kinases through G2/M cell-cycle arrest and a caspase-dependent mechanism.

**Figure 7:**
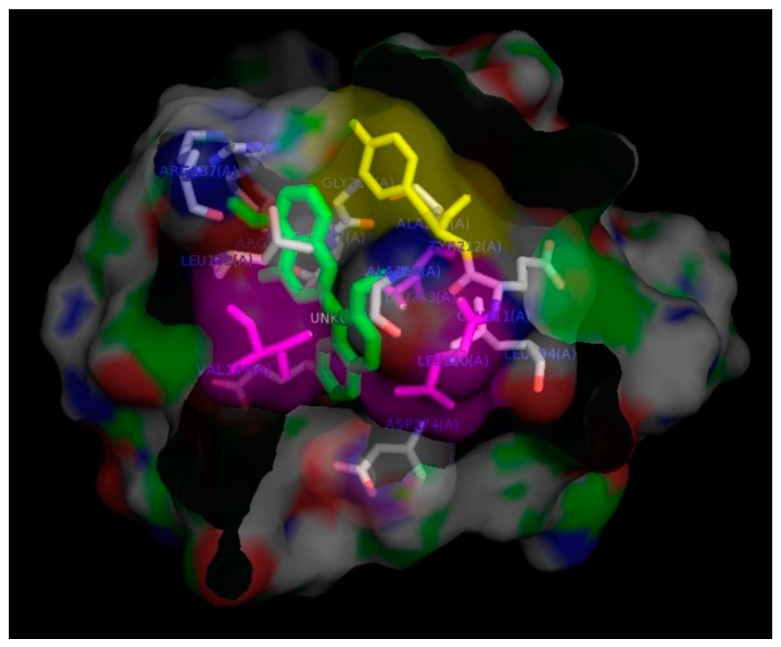
Image of the binding pocket of the AURKA–derivative **31** complex visualized using the PyMol program. Derivative **31** and Tyr212 are colored in green and yellow, respectively. Val147, Leu210 and Leu263 are marked in magenta color.

To elucidate the binding modes between derivative **31** and aurora kinases at the molecular level, in-silico docking experiments were conducted. Among the many X-ray crystallographic structures of AURKA deposited in the protein data bank, 3uod.pdb was selected because its ligand, 4-[(4-[2-(trifluoromethyl)phenyl]amino]pyrimidin-2-yl)amino]benzoic acid (named as TPB) (Figure S7), is more similar to the synthetic flavones used here than the ligands contained in other crystallographic structures deposited in the protein data bank [24]. Its organism, expression system and resolution were *Homo sapiens*, *Escherichia coli* BL21(DE3) and 2.5 Å, respectively. AURKA consists of 403 residues and 3uod.pdb contains residues between Ser123–Lys401, including a kinase domain. This structure contains its ligand as well as 1,2-ethanediol and di(hydroxyethyl)ether. To prepare the apoprotein of 3uod.pdb, its ligand was extracted using the Sybyl/Biopolymer module (Tripos), but 1,2-ethanediol and di(hydroxyethyl)ether were not deleted. The solution structure of the apoprotein was obtained through energy minimization using the Conjugate Gradient algorithm where Tripos force field and Gasteiger-Hückell charges were used. Because comparing this apoprotein with 3uod.pdb resulted in a root-mean-squared deviation value of 0.7 Å, this apoprotein was used for in-silico docking experiments. As mentioned above, the 3D structure of the title compound was determined based on the X-ray crystallographic structure of derivative **18** (2-(2,3-dimethoxynaphthalen-1-yl)-3-hydroxy-6-methoxy-4*H*-chromen-4-one) [15]. The results obtained from AutoDock Vina were visualized using PyMol (The PyMOL Molecular Graphics System, Version 1.0r1; Schrödinger, LLC), and analyzed using LigPlot [25]. The binding pocket of AURKA was analyzed using the Ligplot software, and 14 residues were obtained: Arg137, Leu139, Gly140, Val147, Ala160, Leu194, Glu211, Tyr212, Ala213, Thr217, Arg220, Glu260, Leu263 and Ala213 (Figure S8). The dimensions of the docking box were 16, 8 and 16 for x, y and z, respectively, whereas the centers of x, y and z were 21.494, –21.987 and –10.808, respectively. Because the flexible docking procedure was iterated 30 times, 30 AURKA apoprotein–ligand complexes were generated. Because the original ligand, TPB, was docked into the apoprotein well, in-silico docking of derivative **31** was performed in the same manner as that of the original ligand. Because its binding energies ranged from –9.1 to –7.2 kcal/mol, the thermodynamic stability of the docking process of derivative **31** was considered good for further analysis. The complex with the lowest binding energy was selected. The residues residing in its binding pocket were analyzed using LigPlot: Arg137, Leu139, Val147, Ala160, Leu194, Leu210, Glu211, Tyr212, Ala213, Gly216, Thr217, Arg220, Glu260, Leu263 and Asp274 (Figure S9). The binding pocket was visualized using the PyMol program as shown in Figure 7. Even the AURKA–derivative **31** complex included one more residue in its binding site than the AURKA–TPB complex, and it does not contain hydrogen bonds, unlike the AURKA–TPB complex where two residues, Arg137 and Ala213, participated in hydrogen bonds. The binding pocket around the naphthalene ring of derivative **31** consisted of mainly hydrophobic residues, Val147, Leu210 and Leu263, and the binding pocket was deep and wide enough to hold a naphthyl or resveratrol group. It is also well explained that a bulky group is not favored at the C4′ position because the side chain of Tyr212 could induce steric hindrance with substrates. The results of our analysis of the binding mode of derivative **31** are consistent with the pharmacophores that we proposed.

**Figure 8:**
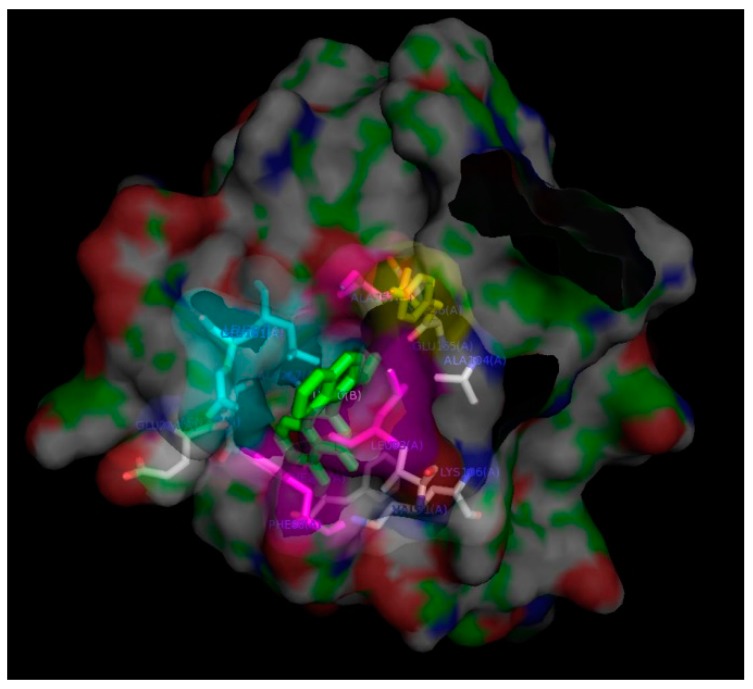
Image of the binding pocket of the AURKB–derivative **31** complex visualized using the PyMol program. Derivative **31** and Tyr156 are colored in green and yellow, respectively. Leu83, Phe88, Ala157 and Leu207 are marked in magenta color. Glu161 is marked in cyan color.

Because Western blotting analysis showed that treatment with derivative **31** decreased the phosphorylation of AURKB, the binding mode between derivative **31** and AURKB was elucidated using in-silico docking in the same manner as that of AURKA. Because 4af3.pdb contained the most residues, it was used for in-silico docking [26]. It originated from *H. sapiens* and was expressed in an *E. coli* BL21 (DE3) system. Its ligand was cyclopropanecarboxylic acid 4-[4-(4-methyl-piperazin-1-yl)-6-(5-methyl-2h-pyrazol-3-ylamino)-pyrimidin-2-ylsulfanyl]-phenyl]-amide (named as VX6). The binding pocket of AURKB was analyzed using Ligplot: Leu83, Phe88, Val91, Ala104, Lys106, Leu138, Glu155, Tyr156, Ala157, Gly160, Glu161, Leu207, Ala217, Asp218 and Phe219 (Figure S10). The dimensions and centers of the docking box were the same as those in the AURKA docking condition. Because the original ligand, VX6, was docked into the apoprotein well, in-silico docking of derivative **31** was performed in the same manner as that of the original ligand. The binding energies of 30 AURKB–derivative **31** complexes ranged from –9.6 to –7.8 kcal/mol, which showed that the complexes were thermodynamically stable. The complex with the lowest binding energy was selected. The residues residing in the binding pocket of the complex were analyzed using LigPlot: Leu83, Phe88, Val91, Ala104, Lys106, Glu155, Tyr156, Ala157, Glu161, Glu204, Asn205, Leu207, Ala217 and Phe219 (Figure S11). The binding pocket was visualized using the PyMol program as shown in Figure 8.

The AURKB–derivative **31** complex contained fewer residues in its binding pocket than the AURKB–VX6 complex. In addition, the AURK–VX6 complex included two hydrogen bonds at Lys106 and Glu155, whereas the AURKB–derivative **31** complex consisted of only hydrophobic interactions. Like the AURKA–derivative **31** complex, the naphthalenyl group is surrounded by hydrophobic residues, Leu83, Phe88, Ala157 and Leu207, and the side chain of Tyr156 resides in the pocket near the naphthalenyl group. However, the hydrophilic residue Glu161 was near the same pocket; thus, the docking of derivative **31** was not favored compared to that of AURKA. The results of Western blotting analysis showed that even though derivative **31** decreased the phosphorylation of both AURKA and AURKB in a dose- and time-dependent manner, the binding modes of derivative **31** to AURKA and AURKB at the molecular level were different from each other.

In conclusion, 36 synthetic flavone derivatives at micromolar concentrations showed half-maximal cell growth inhibitory effects against HCT116 human colon cancer cells. The structural conditions that showed good inhibitory effects on the growth of colon cancer cells were derived based on 3D-QSAR calculations, including the CoMFA and CoMSIA methods, where a bulky group was favored at C2′ and C3′ but was not favored at C4″, a hydrophobic group was favored at C4′, and an electronegative group was not favored at C2′. In our previous study, a flavone derivative inhibited AURKB; thus, Western blotting analysis was performed on derivative **31**, which showed the best half-maximal inhibitory effect on cell growth. Because treatment with derivative **31** decreased the phosphorylation of AURKA, AURKB and AURKC in a dose- and time-dependent manner, this derivative was considered to exhibit pan-aurora kinase inhibitory activity. In addition, flow cytometry results showed that derivative **31** induced apoptosis, and annexin V staining results showed that it triggered apoptosis by inhibiting aurora kinases through G2/M cell-cycle arrest and a caspase-dependent mechanism. The results of binding mode analysis between derivative **31** and AURKA and AURKB at the molecular level using in-silico docking were consistent with the pharmacophores that we proposed. As a result, the synthetic flavone studied here can be developed as a pan-aurora kinase inhibitor and a chemotherapeutic agent.

## 3. Materials and Methods

### 3.1. Preparation of 36 Synthetic Flavone Derivatives

The synthesis and identification of flavone derivatives containing hydroxy, fluoro, bromo, nitro, methoxy, methyl, styryl, and/or naphthalenyl groups were reported previously [7]. The synthetic scheme is provided as Scheme S1 [7]. The names of the derivatives are listed in Table 1. Infrared (IR) spectra were collected using an FT–IR 4200 spectrophotometer (JASCO, Easton, MD, USA) with attenuated total reflection (ATR PR0450-S). IR data as well as the melting points, yields, and purities are provided in the Supplementary Materials. The structures of the derivatives are provided as Table S3. IR spectra are provided in the Supplementary Materials.

### 3.2. Cell Culture

HCT116 human colon cancer cells were obtained from the American Type Culture Collection (Rockville, MD, USA). The cells were maintained in Dulbecco’s modified Eagle’s medium supplemented with 10% fetal bovine serum (CellGro/Corning, Manassas, VA, USA) at 37 °C in a 5% CO_2_ atmosphere [27].

### 3.3. Clonogenic Long-Term Survival Assay

A long-term clonogenic assay was conducted as described previously [11]. Cells were treated with flavone derivatives (0, 5, 10, 20, and 40 μM) for six days. At seven days after treatment, the cells were stained with 0.1% crystal violet. Among the 36 derivatives, 24 derivatives inhibited the growth of the cancer cells almost completely; thus their clonogenicities were measured at lower concentrations (0, 0.1, 0.5, 1, and 5 μM). The inhibitory activities of flavone derivatives on clonogenicity were measured using densitometry (MultiGuage, Fujifilm, Japan), and GI_50_ values were computed using the SigmaPlot software (version 12, SYSTAT, Chicago, IL, USA) [28].

### 3.4. Quantitative Structure–Activity Relationship (QSAR)

Three-dimensional quantitative structure–activity relationship (3D-QSAR) analysis was performed on an Intel Core 2 Quad Q6600 (2.4 GHz) Linux PC with the Sybyl 7.3 software (Tripos, St. Louis, MO, USA) using comparative molecular field analysis (CoMFA) and comparative molecular similarity indices analysis (CoMSIA). The experiment followed previously reported methods [12].

### 3.5. Cell-Cycle Analysis by Flow Cytometry

Cell-cycle status was examined by flow cytometry using propidium iodide [29]. Briefly, HCT116 cells were treated with 5 μM derivative **31** for 0, 12, and 24 h, and fixed in 70% (*v*/*v*) ethanol. Next, the cells were stained with 50 μg/mL propidium iodide solution containing 0.1% (*v*/*v*) Triton X-100, 0.1 mM EDTA, and 50 μg/mL RNase A. Cellular DNA contents were detected by a NucleoCounter NC-3000 cytometer (ChemoMetec, Allerød, Denmark). Diploid (2N) and tetraploid (4N) cells represented cells at the G1 and G2/M phases, respectively. 2N and 4N cells corresponded to those at the S phase. Cells containing DNA lower than 2N DNA were considered as cells at the sub-G1 phase [30].

### 3.6. Apoptosis Assay by Annexin V Staining

Apoptosis assay was performed using a fluorescein isothiocyanate (FITC)-conjugated annexin V kit (BD Pharmingen, San Diego, CA, USA) according to the manufacturer’s instructions. Fluorescence was counted using a NucleoCounter NC-3000 image cytometer (ChemoMetec, Allerød, Denmark) [31].

### 3.7. Western Blotting Analysis

HCT116 cells were treated with derivative **31** for the indicated times. Cell lysates were prepared and immunoblotted according to standard procedures. Antibody-reactive protein bands were visualized using an enhanced chemiluminescence detection system (GE Healthcare, Piscataway, NJ, USA). Antibodies against phospho-aurora kinase A (T288)/aurora kinase B (T232)/aurora kinase C (T198), cleaved caspase-7 (Asp198), and poly(ADP-ribose) polymerase (PARP) were obtained from Cell Signaling Technology (Beverly, MA, USA). Antibodies specific to GAPDH were obtained from Santa Cruz Biotechnology (Santa Cruz, CA, USA) [6].

### 3.8. In-Silico Docking

To elucidate the molecular binding modes between the title compound and aurora kinases, in-silico docking was conducted using AutoDock Vina. In addition, preparation of holoproteins and apoproteins, as well as determination of binding site were performed using the Sybyl program (Tripos) [32]. The 3D structures of aurora kinases were obtained from the protein databank. The experiments followed previously reported methods [27].

### 3.9. Statistical Analysis

Statistical significance was analyzed using Student′s *t*-test [6]. A *p*-value of less than 0.05 was considered statistically significant. All experiments were performed in triplicate.

## Figures and Tables

**Table 1:** Names of synthetic flavone derivatives **1**–**36**, and their half-maximal cell growth inhibitory effect (GI_50_) and the negative logarithmic scales of GI_50_ values (pGI_50_).

Derivatives	Chemical Names	GI_50_ (μM)	pGI_50_
**1**	2-(2-fluorophenyl)-3-hydroxy-4*H*-chromen-4-one/2′-fluoroflavone	41.19	1.39
**2**	2-(2-fluorophenyl)-3-hydroxy-6-nitro-4*H*-chromen-4-one/2′-fluoro-6-nitroflavone	33.52	1.47
**3**	2-(4-fluorophenyl)-3-hydroxy-6-nitro-4*H*-chromen-4-one/4′-fluoro-6-nitroflavone	4.49	2.35
**4**	3-hydroxy-2-(4-methoxyphenyl)-4*H*-chromen-4-one/4′-methoxyflavone	37.18	1.43
**5**	3-hydroxy-2-(2-methoxyphenyl)-4*H*-chromen-4-one/2′-methoxyflavone	16.44	1.78
**6**	2-(3,4-dimethoxyphenyl)-3-hydroxy-4*H*-chromen-4-one/3′,4′-dimethoxyflavone	3.59	2.44
**7**	3-hydroxy-2-(2,4,6-trimethoxyphenyl)-4*H*-chromen-4-one/2′,4′,6′-trimethoxyflavone	4.53	2.34
**8**	2-(2,4-dimethoxyphenyl)-3-hydroxy-4*H*-chromen-4-one/2′,4′-dimethoxyflavone	21.92	1.66
**9**	2-(6-(4-methoxystyryl)-2,4-dimethoxyphenyl)-3-hydroxy-4*H*-chromen-4-one/3-hydroxy-2′-(4-methoxystyryl)-flavone	3.18	2.50
**10**	2-(6-(4-methoxystyryl)-2,4-dimethoxyphenyl)-3-hydroxy-6-nitro-4*H*-chromen-4-one/3-hydroxy-6-nitro-2′-(4-methoxystyryl)-flavone	3.63	2.44
**11**	2-(6-(4-methoxystyryl)-2,4-dimethoxyphenyl)-6-bromo-3-hydroxy-4*H*-chromen-4-one/3-hydroxy-6-bromo-2′-(4-methoxystyryl)-flavone	2.66	2.58
**12**	2-(6-(4-methoxystyryl)-2,4-dimethoxyphenyl)-7-fluoro-3-hydroxy-4*H*-chromen-4-one/7-fluoro-3-hydroxy-2′-(4-methoxystyryl)-flavone	3.07	2.51
**13**	2-(6-(4-methoxystyryl)-2,4-dimethoxyphenyl)-6-chloro-3-hydroxy-4*H*-chromen-4-one/3-hydroxy-6-chloro-2′-(4-methoxystyryl)-flavone	2.87	2.54
**14**	2-(6-(4-methoxystyryl)-2,4-dimethoxyphenyl)-6-fluoro-3-hydroxy-4*H*-chromen-4-one/3-hydroxy-6-fluoro-2′-(4-methoxystyryl)-flavone	2.41	2.62
**15**	3-hydroxy-2-(naphthalen-1-yl)-4*H*-chromen-4-one/3-hydroxy-2′,3′-naphthoflavone	4.14	2.38
**16**	3-hydroxy-6-methoxy-2-(naphthalen-1-yl)-4*H*-chromen-4-one/3-hydroxy-6-methoxy-2′,3′-naphthoflavone	7.21	2.14
**17**	3-hydroxy-2-(2-methoxynaphthalen-1-yl)-4*H*-chromen-4-one/3-hydroxy-6′-methoxy-2′,3′-naphthoflavone	5.28	2.28
**18**	2-(2,3-dimethoxynaphthalen-1-yl)-3-hydroxy-6-methoxy-4*H*-chromen-4-one/3-hydroxy-5′,6,6′-trimethoxy-2′,3′-naphthoflavone	7.51	2.12
**19**	3-hydroxy-2-(4-methoxynaphthalen-1-yl)-4*H*-chromen-4-one/3-hydroxy-4′-methoxy-2′,3′-naphthoflavone	4.28	2.37
**20**	3-hydroxy-2-(naphthalen-2-yl)-4*H*-chromen-4-one/3-hydroxy-3′,4′-naphthoflavone	2.41	2.62
**21**	3-hydroxy-6-methoxy-2-(naphthalen-2-yl)-4*H*-chromen-4-one/3-hydroxy-6-methoxy-3′,4′-naphthoflavone	3.07	2.51
**22**	2-(naphthalen-1-yl)-4*H*-chromen-4-one/2′,3′-naphthoflavone	2.91	2.54
**23**	6-methoxy-2-(naphthalen-1-yl)-4*H*-chromen-4-one/6-methoxy-2′,3′-naphthoflavone	2.41	2.62
**24**	5-methoxy-2-(naphthalen-1-yl)-4*H*-chromen-4-one/5-methoxy-2′,3′-naphthoflavone	7.31	2.14
**25**	6,7-dimethoxy-2-(naphthalen-1-yl)-4*H*-chromen-4-one/6,7-dimethoxy-2′,3′-naphthoflavone	3.26	2.49
**26**	7-methoxy-2-(naphthalen-1-yl)-4*H*-chromen-4-one/7-methoxy-2′,3′-naphthoflavone	2.56	2.59
**27**	2-(naphthalen-2-yl)-4*H*-chromen-4-one/3′,4′-naphthoflavone	29.86	1.52
**28**	6-methoxy-2-(naphthalen-2-yl)-4*H*-chromen-4-one/6-methoxy-3′,4′-naphthoflavone	24.39	1.61
**29**	2-(2-methoxynaphthalen-1-yl)-4*H*-chromen-4-one/2′-methoxy-2′,3′-naphthoflavone	4.06	2.39
**30**	6-methoxy-2-(2-methoxynaphthalen-1-yl)-4*H*-chromen-4-one/2′,6-dimethoxy-2′,3′-naphthoflavone	2.78	2.56
**31**	5-methoxy-2-(2-methoxynaphthalen-1-yl)-4*H*-chromen-4-one/2′,5-dimethoxy-2′,3′-naphthoflavone	0.49	3.31
**32**	6,7-dimethoxy-2-(2-methoxynaphthalen-1-yl)-4*H*-chromen-4-one/2′,6,7-trimethoxy-2′,3′-naphthoflavone	3.80	2.42
**33**	2-(4-methoxynaphthalen-1-yl)-4*H*-chromen-4-one/4′-methoxy-2′,3′-naphthoflavone	20.80	1.68
**34**	5,7-dimethoxy-2-(4-methoxynaphthalen-1-yl)-4*H*-chromen-4-one/4′,5,7-trimethoxy-2′,3′-naphthoflavone	21.56	1.67
**35**	7-methoxy-2-(4-methoxynaphthalen-1-yl)-4*H*-chromen-4-one/4′,7-dimethoxy-2′,3′-naphthoflavone	18.18	1.74
**36**	2-(2,3-dimethoxynaphthalen-1-yl)-7-methoxy-4*H*-chromen-4-one/2′,3′,7-trimethoxy-2′,3′-naphthoflavone	3.95	2.40

## Supplementary Materials

Supplementary materials can be found at http://www.mdpi.com/1422-0067/19/12/4086/s1.

## Abbreviations

GI_50_	cell growth inhibitory concentration
AURKA	aurora kinase A
AURKB	aurora kinase B
AURKC	aurora kinase C
CoMFA	comparative molecular field analysis
CoMSIA	comparative molecular similarity indices analysis
3D-QSAR	Three-dimensional quantitative structure–activity relationship
PARP	poly(ADP-ribose) polymerase
TPB	4-[(4-[2-(trifluoromethyl)phenyl]amino]pyrimidin-2-yl)amino]benzoic acid
FITC	fluorescein isothiocyanate
