# Supplementary material for: Inhibitory Effect of Synthetic Flavone Derivatives on Pan-Aurora Kinases: Induction of G2/M Cell-Cycle Arrest and Apoptosis in HCT116 Human Colon Cancer Cells
Source: Int J Mol Sci. 2018 Dec 17;19(12):4086. doi: 10.3390/ijms19124086 (PMC6320900; doi:10.3390/ijms19124086)

The melting points, yields, purities, and IR data of the 36 flavone derivatives

2-(2-fluorophenyl)-3-hydroxy-4*H*-chromen-4-one (**1**)
Yield: 56%; m.p. 150-156°C; purity: 98%; IR (v/cm^{-1}) v (C=O, conjugated) : 1606, v (OH) : 3298

2-(2-fluorophenyl)-3-hydroxy-6-nitro-4*H*-chromen-4-one (**2**)
Yield: 96%; m.p. 194-200°C; purity: 98%; IR (v/cm^{-1}) v (C=O, conjugated) : 1622, v (OH) : 3289

2-(4-fluorophenyl)-3-hydroxy-6-nitro-4*H*-chromen-4-one (**3**)
Yield: 33%; m.p. 240-244°C; purity: 96%; IR (v/cm^{-1}) v (C=O, conjugated) : 1612, v (OH) : 3284

3-hydroxy-2-(4-methoxyphenyl)-4*H*-chromen-4-one (**4**)
Yield: 60%; m.p. 234-238°C; purity: 98%; IR (v/cm^{-1}) v (C=O, conjugated) : 1602, v (OH) : 3189

3-hydroxy-2-(2-methoxyphenyl)-4*H*-chromen-4-one (**5**)
Yield: 62%; m.p. 210-212°C; purity: 98%; IR (v/cm^{-1}) v (C=O, conjugated) : 1607, v (OH) : 3286

2-(3,4-dimethoxyphenyl)-3-hydroxy-4*H*-chromen-4-one (**6**)
Yield: 57%; m.p. 200-202°C; purity: 98%; IR (v/cm^{-1}) v (C=O, conjugated) : 1610, v (OH) : 3211

3-hydroxy-2-(2,4,6-trimethoxyphenyl)-4*H*-chromen-4-one (**7**)
Yield: 62%; m.p. 190-194°C; purity: 98%; IR (v/cm^{-1}) v (C=O, conjugated) : 1605, v (OH) : 3191

2-(2,4-dimethoxyphenyl)-3-hydroxy-4*H*-chromen-4-one (**8**)
Yield: 64%; m.p. 198-200°C; purity: 97%; IR (v/cm^{-1}) v (C=O, conjugated) : 1609, v (OH) : 3306

2-(6-(4-methoxystyryl)-2,4-dimethoxyphenyl)-3-hydroxy-4*H*-chromen-4-one (**9**)
Yield: 46%; m.p. 206-210°C; purity: 98%; IR (v/cm^{-1}) v (C=O, conjugated) : 1610, v (OH) : 3314

2-(6-(4-methoxystyryl)-2,4-dimethoxyphenyl)-3-hydroxy-6-nitro-4*H*-chromen-4-one (**10**)
Yield: 78%; m.p. 118-122°C; purity: 97%; IR (v/cm^{-1}) v (C=O, conjugated) : 1620, v (OH) : 3323

2-(6-(4-methoxystyryl)-2,4-dimethoxyphenyl)-6-bromo-3-hydroxy-4*H*-chromen-4-one (**11**)
Yield: 71%; m.p. 161-168°C; purity: 94%; IR (v/cm^{-1}) v (C=O, conjugated) : 1599, v (OH) : 3303

2-(6-(4-methoxystyryl)-2,4-dimethoxyphenyl)-7-fluoro-3-hydroxy-4*H*-chromen-4-one (**12**)

Yield: 80%; m.p. 228-230°C; purity: 98%; IR (*v*/cm^{-1}) *v* (C=O, conjugated) : 1606, *v* (OH) : 3289

2-(6-(4-methoxystyryl)-2,4-dimethoxyphenyl)-6-chloro-3-hydroxy-4*H*-chromen-4-one (**13**)
Yield: 56%; m.p. 180-182°C; purity: 98%; IR (*v*/cm^{-1}) *v* (C=O, conjugated) : 1598, *v* (OH) : 3314

2-(6-(4-methoxystyryl)-2,4-dimethoxyphenyl)-6-fluoro-3-hydroxy-4*H*-chromen-4-one (**14**)
Yield: 87%; m.p. 196-200°C; purity: 98%; IR (*v*/cm^{-1}) *v* (C=O, conjugated) : 1603, *v* (OH) : 3328

3-hydroxy-2-(naphthalen-1-yl)-4*H*-chromen-4-one (**15**)
Yield: 32%; m.p. 202-204°C; purity: 98%; IR (*v*/cm^{-1}) *v* (C=O, conjugated) : 1602, *v* (OH) : 3358

3-hydroxy-6-methoxy-2-(naphthalen-1-yl)-4*H*-chromen-4-one (**16**)
Yield: 31%; m.p. 220-226°C; purity: 96%; IR (*v*/cm^{-1}) *v* (C=O, conjugated) : 1605, *v* (OH) : 3290

3-hydroxy-2-(2-methoxynaphthalen-1-yl)-4*H*-chromen-4-one (**17**)
Yield: 94%; m.p. 240-242°C; purity: 98%; IR (*v*/cm^{-1}) *v* (C=O, conjugated) : 1608, *v* (OH) : 3278

2-(2,3-dimethoxynaphthalen-1-yl)-3-hydroxy-6-methoxy-4*H*-chromen-4-one (**18**)
Yield: 64%; m.p. 280-284°C; purity: 98%; IR (*v*/cm^{-1}) *v* (C=O, conjugated) : 1596, *v* (OH) : 3331

3-hydroxy-2-(4-methoxynaphthalen-1-yl)-4*H*-chromen-4-one (**19**)
Yield: 79%; m.p. 230-232°C; purity: 98%; IR (*v*/cm^{-1}) *v* (C=O, conjugated) : 1617, *v* (OH) : 3339

3-hydroxy-2-(naphthalen-2-yl)-4*H*-chromen-4-one (**20**)
Yield: 50%; m.p. 190-192°C; purity: 98%; IR (*v*/cm^{-1}) *v* (C=O, conjugated) : 1605, *v* (OH) : 3251

3-hydroxy-6-methoxy-2-(naphthalen-2-yl)-4*H*-chromen-4-one (**21**)
Yield: 40%; m.p. 200-203°C; purity: 98%; IR (*v*/cm^{-1}) *v* (C=O, conjugated) : 1599, *v* (OH) : 3320

2-(naphthalen-1-yl)-4*H*-chromen-4-one (**22**)
Yield: 84%; m.p. 117-122°C; purity: 98%; IR (*v*/cm^{-1}) *v* (C=O, conjugated) : 1651, *v* (CH, sp^2) : 3052

6-methoxy-2-(naphthalen-1-yl)-4*H*-chromen-4-one (**23**)
Yield: 39%; m.p. 108-112°C; purity: 98%; IR (*v*/cm^{-1}) *v* (C=O, conjugated) : 1640, *v* (CH, sp^2) : 3052

5-methoxy-2-(naphthalen-1-yl)-4*H*-chromen-4-one (**24**)

Yield: 50%; m.p. 202-206°C; purity: 96%; IR (*v*/cm^{-1}) *v* (C=O, conjugated) : 1641, *v* (CH, sp^2) : 3049

6,7-dimethoxy-2-(naphthalen-1-yl)-4*H*-chromen-4-one (**25**)

Yield: 45%; m.p. 170-176°C; purity: 98%; IR (*v*/cm^{-1}) *v* (C=O, conjugated) : 1629, *v* (CH, sp^2) : 3004

7-methoxy-2-(naphthalen-1-yl)-4*H*-chromen-4-one (**26**)

Yield: 10%; m.p. 102-106°C; purity: 98%; IR (*v*/cm^{-1}) *v* (C=O, conjugated) : 1639, *v* (CH, sp^2) : 3057

2-(naphthalen-2-yl)-4*H*-chromen-4-one (**27**)

Yield: 24%; m.p. 158°C; purity: 96%; IR (*v*/cm^{-1}) *v* (C=O, conjugated) : 1622, *v* (CH, sp^2) : 3054

6-methoxy-2-(naphthalen-2-yl)-4*H*-chromen-4-one (**28**)

Yield: 70%; m.p. 210-214°C; purity: 97%; IR (*v*/cm^{-1}) *v* (C=O, conjugated) : 3057, *v* (CH, sp^2) : 1635

2-(2-methoxynaphthalen-1-yl)-4*H*-chromen-4-one (**29**)

Yield: 81%; m.p. 200-202°C; purity: 98%; IR (*v*/cm^{-1}) *v* (C=O, conjugated) : 3052, *v* (CH, sp^2) : 1642

6-methoxy-2-(2-methoxynaphthalen-1-yl)-4*H*-chromen-4-one (**30**)

Yield: 44%; m.p. 187-189°C; purity: 98%; IR (*v*/cm^{-1}) *v* (C=O, conjugated) : 1637, *v* (CH, sp^2) : 3067

5-methoxy-2-(2-methoxynaphthalen-1-yl)-4*H*-chromen-4-one (**31**)

Yield: 85%; m.p. 74-78°C; purity: 98%; IR (*v*/cm^{-1}) *v* (C=O, conjugated) : 1642, *v* (CH, sp^2) : 3060

6,7-dimethoxy-2-(2-methoxynaphthalen-1-yl)-4*H*-chromen-4-one (**32**)

Yield: 22%; m.p. 226-230°C; purity: 98%; IR (*v*/cm^{-1}) *v* (C=O, conjugated) : 1622, *v* (CH, sp^2) : 3067

2-(4-methoxynaphthalen-1-yl)-4*H*-chromen-4-one (**33**)

Yield: 59%; m.p. 175-180°C; purity: 97%; IR (*v*/cm^{-1}) *v* (C=O, conjugated) : 1649, *v* (CH, sp^2) : 3065

5,7-dimethoxy-2-(4-methoxynaphthalen-1-yl)-4*H*-chromen-4-one (**34**)

Yield: 98%; m.p. 202-208°C; purity: 97%; IR (*v*/cm^{-1}) *v* (C=O, conjugated) : 1641, *v* (CH, sp^2) : 3074

7-methoxy-2-(4-methoxynaphthalen-1-yl)-4*H*-chromen-4-one (**35**)

Yield: 22%; m.p. 184-188°C; purity: 98%; IR (*v*/cm^{-1}) *v* (C=O, conjugated) : 1637, *v* (CH, sp^2) : 3084

2-(2,3-dimethoxynaphthalen-1-yl)-7-methoxy-4*H*-chromen-4-one (**36**)

Yield: 95%; m.p. 154-160°C; purity: 95%; IR (*v*/cm^{-1}) *v* (C=O, conjugated) : 1640, *v* (CH, sp^2) : 3059

Supp. Table 1. A comparison of the pGI_{50} values obtained from the clonogenic long-term survival assay with the values predicted using the CoMFA model, and their residual values. * denotes the derivatives contained in the test set.

derivatives	Experimental data (pGI_{50})	Predicted value (pGI_{50})	Residual (%)
**1**	1.39	1.46	-5.08
**2**	1.47	1.60	-8.18
**3**	2.35	2.28	2.89
**4**	1.43	1.36	4.99
**5**	1.78	1.82	-2.02
**6**	2.44	2.50	-2.32
**7***	2.34	2.19	6.80
**8**	1.66	1.63	1.99
**9**	2.50	2.51	-0.49
**10***	2.44	2.64	-8.01
**11**	2.58	2.57	0.29
**12**	2.51	2.50	0.40
**13**	2.54	2.54	0.11
**14***	2.62	2.44	6.79
**15**	2.38	2.26	5.12
**16**	2.14	2.19	-2.39
**17***	2.28	2.85	-24.96
**18**	2.12	2.15	-1.40
**19**	2.37	2.38	-0.67
**20**	2.62	2.61	0.24
**21**	2.51	2.56	-1.65
**22***	2.54	2.06	18.69
**23**	2.62	2.40	8.29
**24**	2.14	2.17	-1.44
**25**	2.49	2.56	-2.82
**26**	2.59	2.59	0.13
**27**	1.52	1.57	-2.74
**28**	1.61	1.55	3.81
**29***	2.39	2.70	-12.81
**30**	2.56	2.61	-2.23
**31**	3.31	3.31	-0.14
**32***	2.42	2.81	-15.99
**33**	1.68	1.65	1.68
**34**	1.67	1.59	4.53
**35**	1.74	1.83	-5.15
**36**	2.40	2.38	0.89

Supp. Table 2. A comparison of the pGI_{50} values obtained from the clonogenic long-term survival assay with the values predicted using the CoMSIA model, and their residual values. * denotes the derivatives contained in the test set.

derivatives	Experimental data (pGI_{50})	Predicted value (pGI_{50})	Residual (%)
**1**	1.39	1.30	6.01
**2**	1.47	1.59	-7.80
**3**	2.35	2.19	6.56
**4**	1.43	1.40	2.21
**5**	1.78	1.75	1.70
**6**	2.44	2.55	-4.35
**7***	2.34	1.84	21.57
**8**	1.66	1.55	6.76
**9**	2.50	2.56	-2.40
**10***	2.44	2.84	-16.44
**11**	2.58	2.49	3.51
**12**	2.51	2.61	-3.92
**13**	2.54	2.49	2.17
**14***	2.62	2.43	7.34
**15**	2.38	2.37	0.58
**16**	2.14	2.33	-8.82
**17***	2.28	2.82	-23.92
**18**	2.12	2.17	-2.12
**19**	2.37	2.26	4.76
**20**	2.62	2.61	0.18
**21**	2.51	2.59	-3.14
**22***	2.54	2.10	17.18
**23**	2.62	2.36	9.76
**24**	2.14	2.13	0.42
**25**	2.49	2.44	1.72
**26**	2.59	2.54	2.13
**27**	1.52	1.69	-10.62
**28**	1.61	1.70	-5.35
**29***	2.39	2.70	-12.71
**30**	2.56	2.71	-5.99
**31**	3.31	3.24	2.11
**32***	2.42	2.78	-14.91
**33**	1.68	1.70	-0.80
**34**	1.67	1.61	3.26
**35**	1.74	1.88	-7.77
**36**	2.40	2.34	2.82

Suppl. Table 3. Structures of the 36 flavone derivatives.

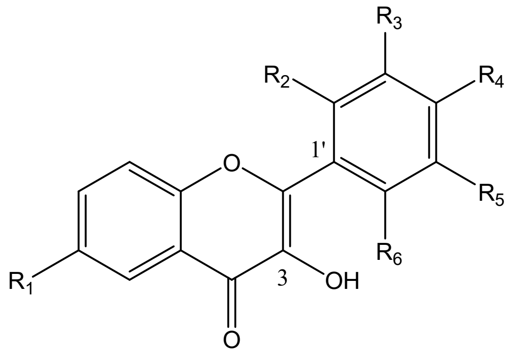

derivative	R_1	R_2	R_3	R_4	R_5	R_6
**1**	H	F	H	H	H	H
**2**	NO_2	F	H	H	H	H
**3**	NO_2	H	H	F	H	H
**4**	H	H	H	OCH_3	H	H
**5**	H	OCH_3	H	H	H	H
**6**	H	H	OCH_3	OCH_3	H	H
**7**	H	OCH_3	H	OCH_3	H	OCH_3
**8**	H	OCH_3	H	OCH_3	H	H

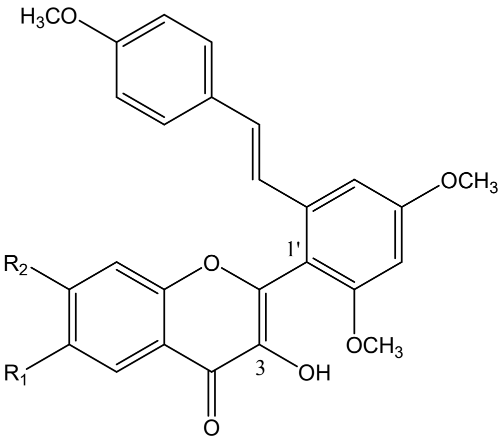

derivative	R_1	R_2
**9**	H	H
**10**	NO_2	H
**11**	Br	H
**12**	H	F
**13**	Cl	H
**14**	F	H

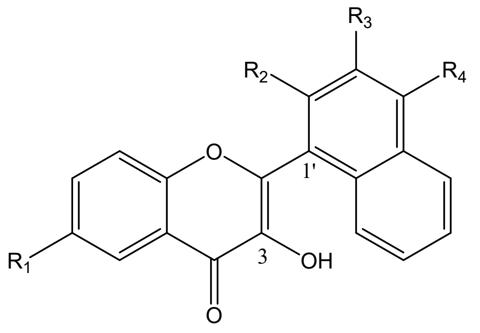

derivative	R_1	R_2	R_3	R_4
**15**	H	H	H	H
**16**	OCH_3	H	H	H
**17**	H	OCH_3	H	H
**18**	OCH_3	OCH_3	OCH_3	H
**19**	H	H	H	OCH_3

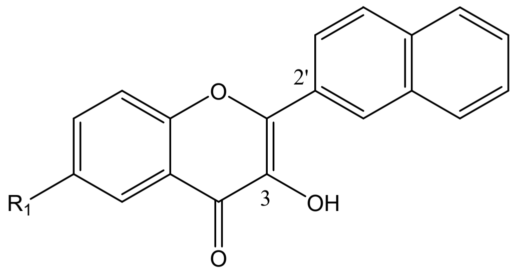

derivative	R_1
**20**	H
**21**	OCH_3

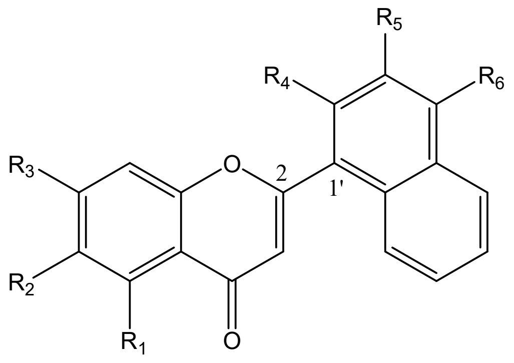

derivative	R_1	R_2	R_3	R_4	R_5	R_6
**22**	H	H	H	H	H	H
**23**	H	OCH_3	H	H	H	H
**24**	OCH_3	H	H	H	H	H
**25**	H	OCH_3	OCH_3	H	H	H
**26**	H	H	OCH_3	H	H	H
**29**	H	H	H	OCH_3	H	H
**30**	H	OCH_3	H	OCH_3	H	H
**31**	OCH_3	H	H	OCH_3	H	H
**32**	H	OCH_3	OCH_3	OCH_3	H	H
**33**	H	H	H	H	H	OCH_3
**34**	OCH_3	H	OCH_3	H	H	OCH_3
**35**	H	H	OCH_3	H	H	OCH_3
**36**	H	H	OCH_3	OCH_3	OCH_3	H

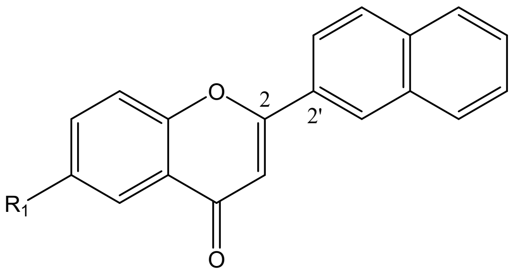

derivative	R_1
**27**	H
**28**	OCH_3

Suppl. Figure 1. Hierarchical clustering analysis used to select the test set.

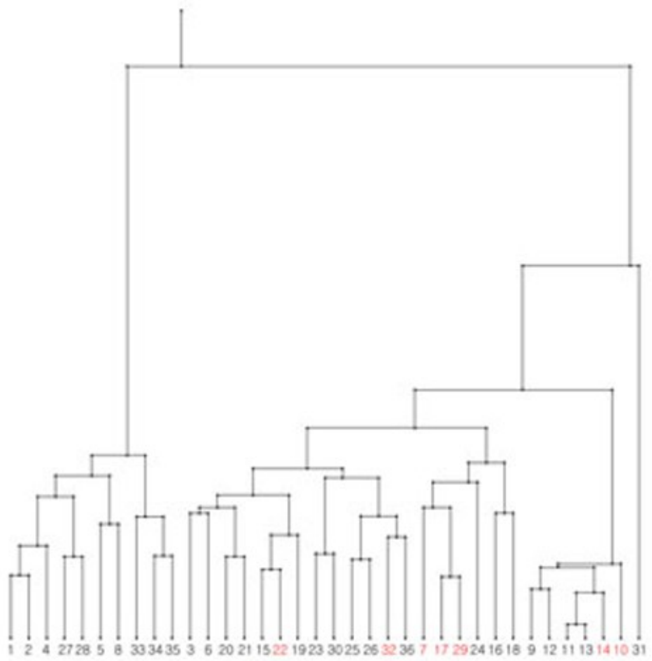

Suppl. Figure 2. Alignments of 29 derivatives included in the training set.

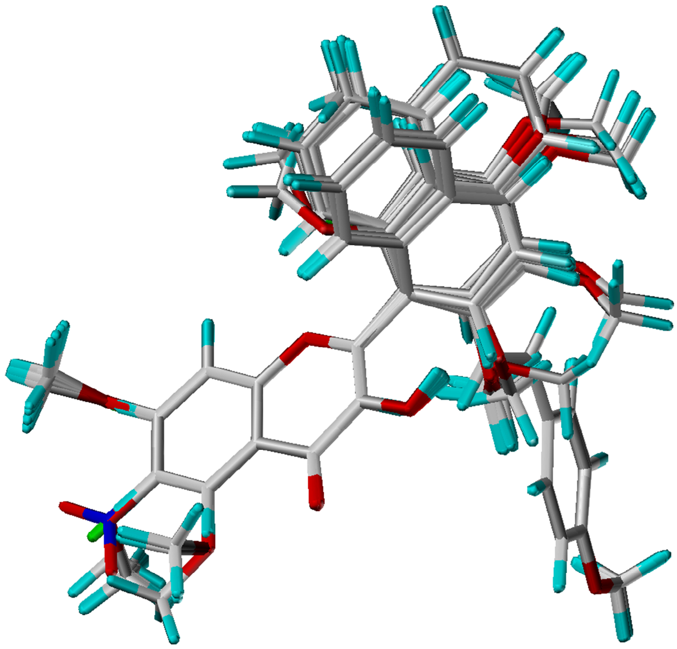

Suppl. Figure 3. A comparison of the experimental pGI_{50} values obtained from the clonogenic long-term survival assay with the values predicted using the CoMFA model. The training set and the test set are denoted in blue and red squares, respectively.

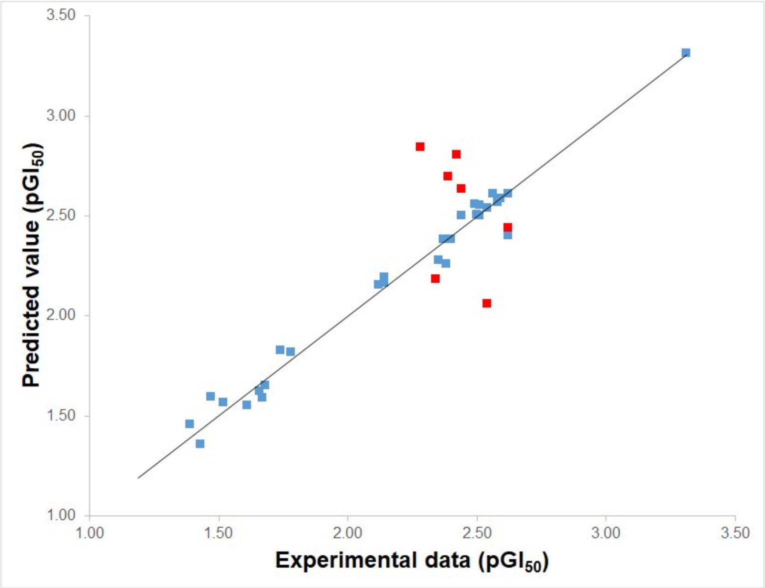

Suppl. Figure 4. (A) Contour maps of steric field descriptors generated by the CoMFA model. The bulk favoring (green) and disfavoring regions (yellow) occupied 92% and 8%, respectively. (B) Contour maps of electrostatic field descriptors. The electrostatic favoring (blue) and disfavoring regions (red) occupied 1% and 99% of the maps, respectively.

(A)

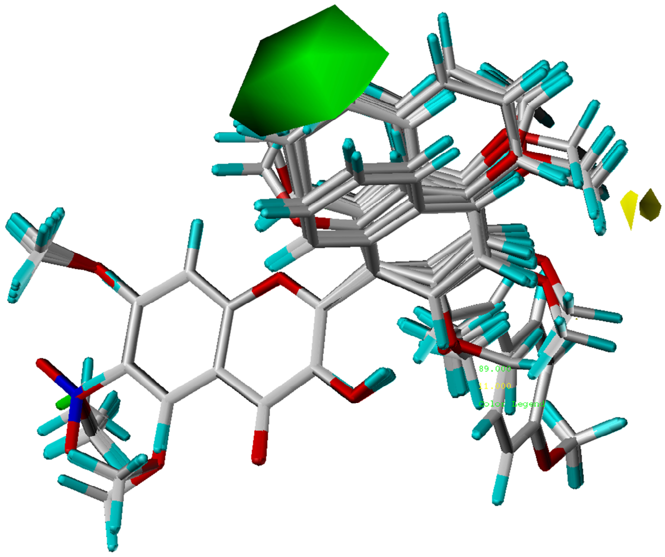

(B)

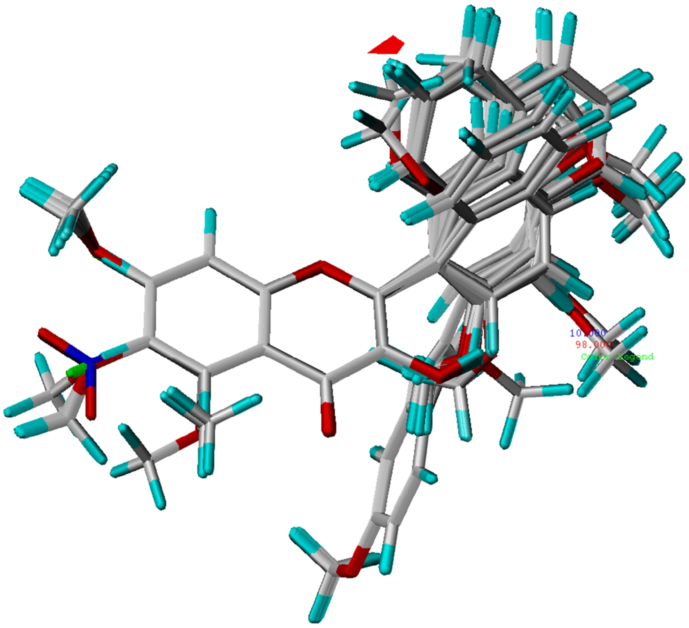

Suppl. Figure 5. A comparison of the experimental pGI_{50} values obtained from the clonogenic long-term survival assay with the values predicted using the CoMSIA model. The training set and the test set are denoted in blue and red squares, respectively.

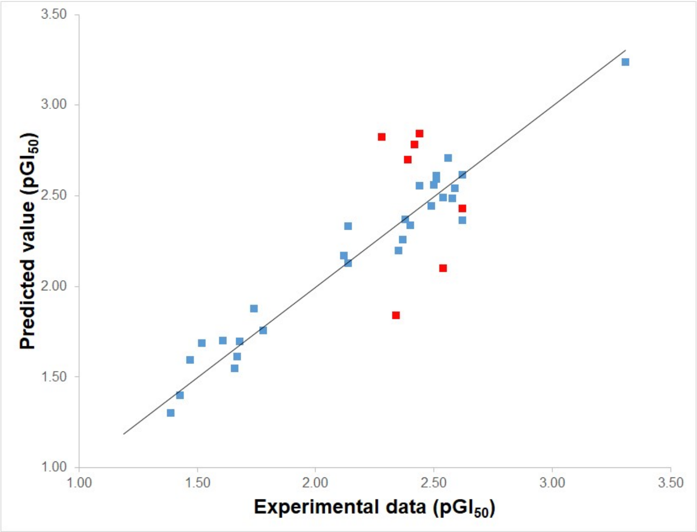

Suppl. Figure 6. Contour maps of hydrophobic field descriptors generated by the CoMSIA model. The hydrophobic favoring region (orange) occupied 95% and the disfavoring region (violet) 5%, of the contour map area.

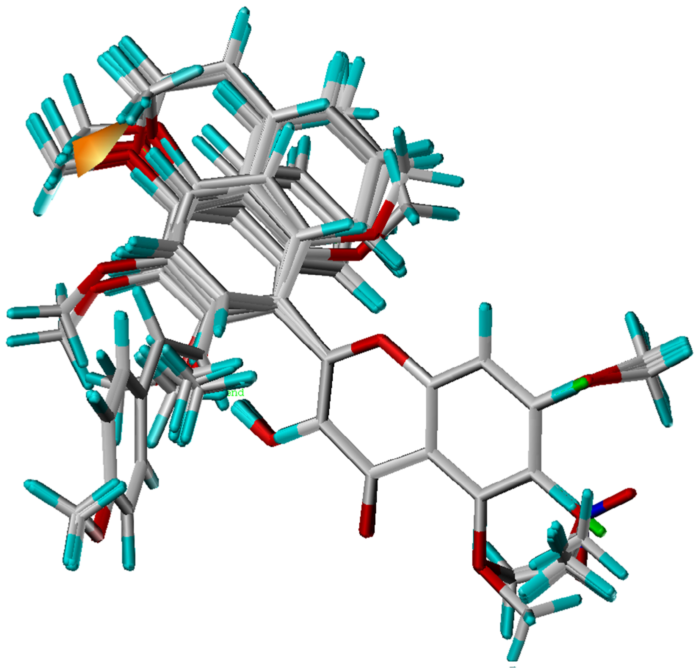

Suppl. Figure 7. The structure of the ligand contained in AURKA deposited in the protein data bank as 3uod.pdb, 4-[(4-{[2-(trifluoromethyl)phenyl]amino}pyrimidin-2-yl)amino]benzoic acid.

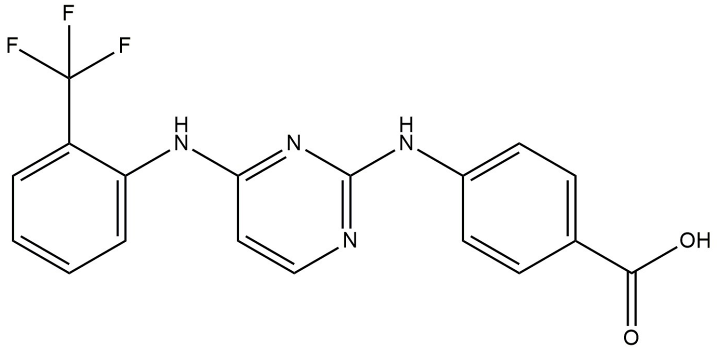

Suppl. Figure 8. Residues residing in the binding site of the AURKA – TPB complex obtained by the LigPlot analysis.

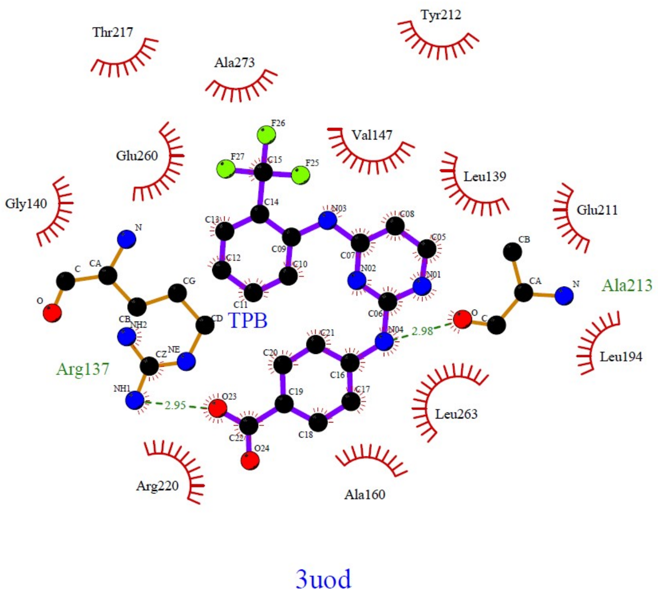

Suppl. Figure 9. Residues residing in the binding site of the AURKA – derivative **31** complex obtained by the LigPlot analysis.

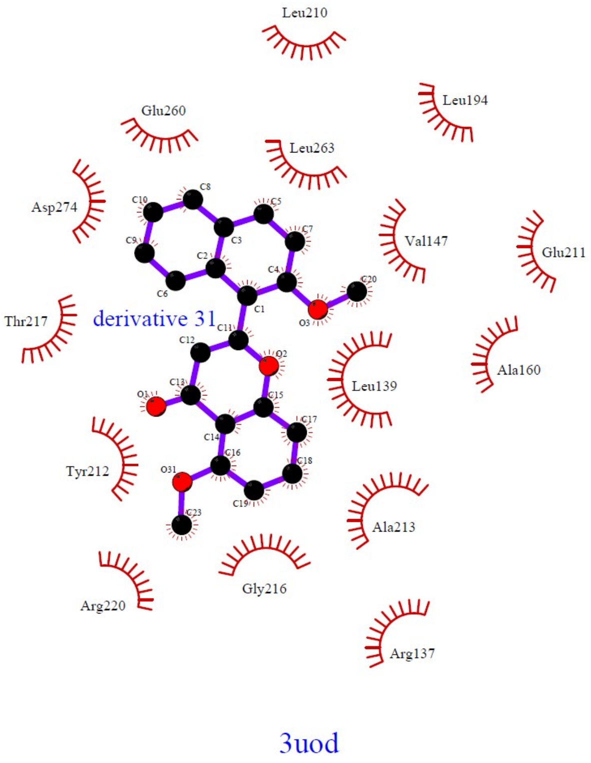

Suppl. Figure 10. Residues residing in the binding site of the AURKB – VX6 complex obtained by the LigPlot analysis.

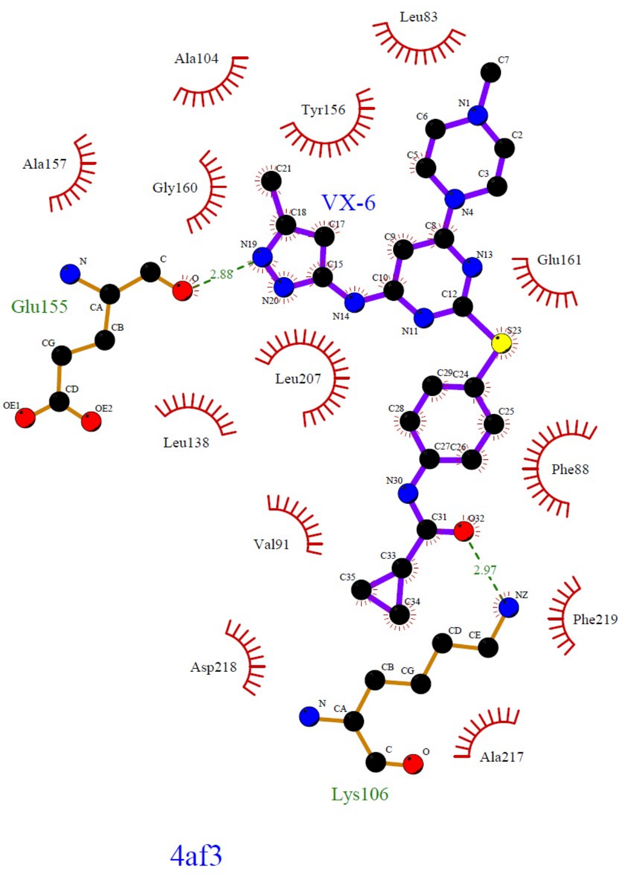

Suppl. Figure 11. Residues residing in the binding site of the AURKB – derivative **31** complex obtained by the LigPlot analysis.

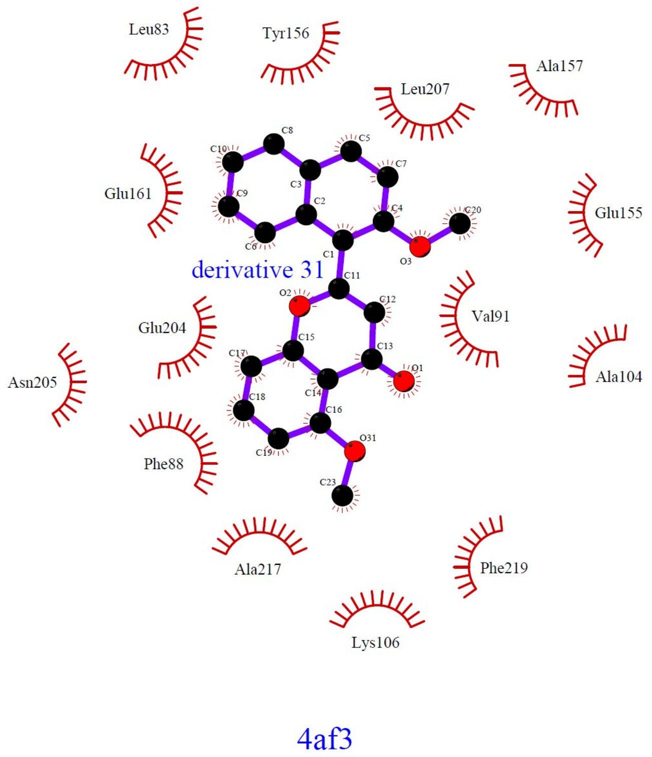

Suppl. Scheme 1. The synthetic procedure of 36 flavone derivatives [*Magn Reson Chem* **2017,** 55, 359].

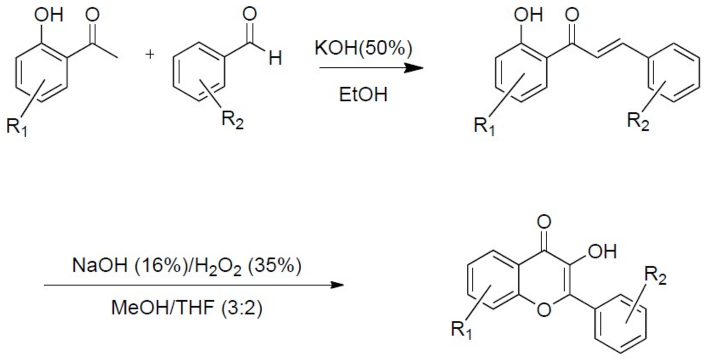

Supplement: Supplementary file 1 [file ijms-19-04086-s001.zip › supplementary materials.pdf]
