# Supplementary material for: Inhibitory Effect of Synthetic Flavone Derivatives on Pan-Aurora Kinases: Induction of G2/M Cell-Cycle Arrest and Apoptosis in HCT116 Human Colon Cancer Cells
Source: Int J Mol Sci. 2018 Dec 17;19(12):4086. doi: 10.3390/ijms19124086 (PMC6320900; doi:10.3390/ijms19124086)

Derivative **1**

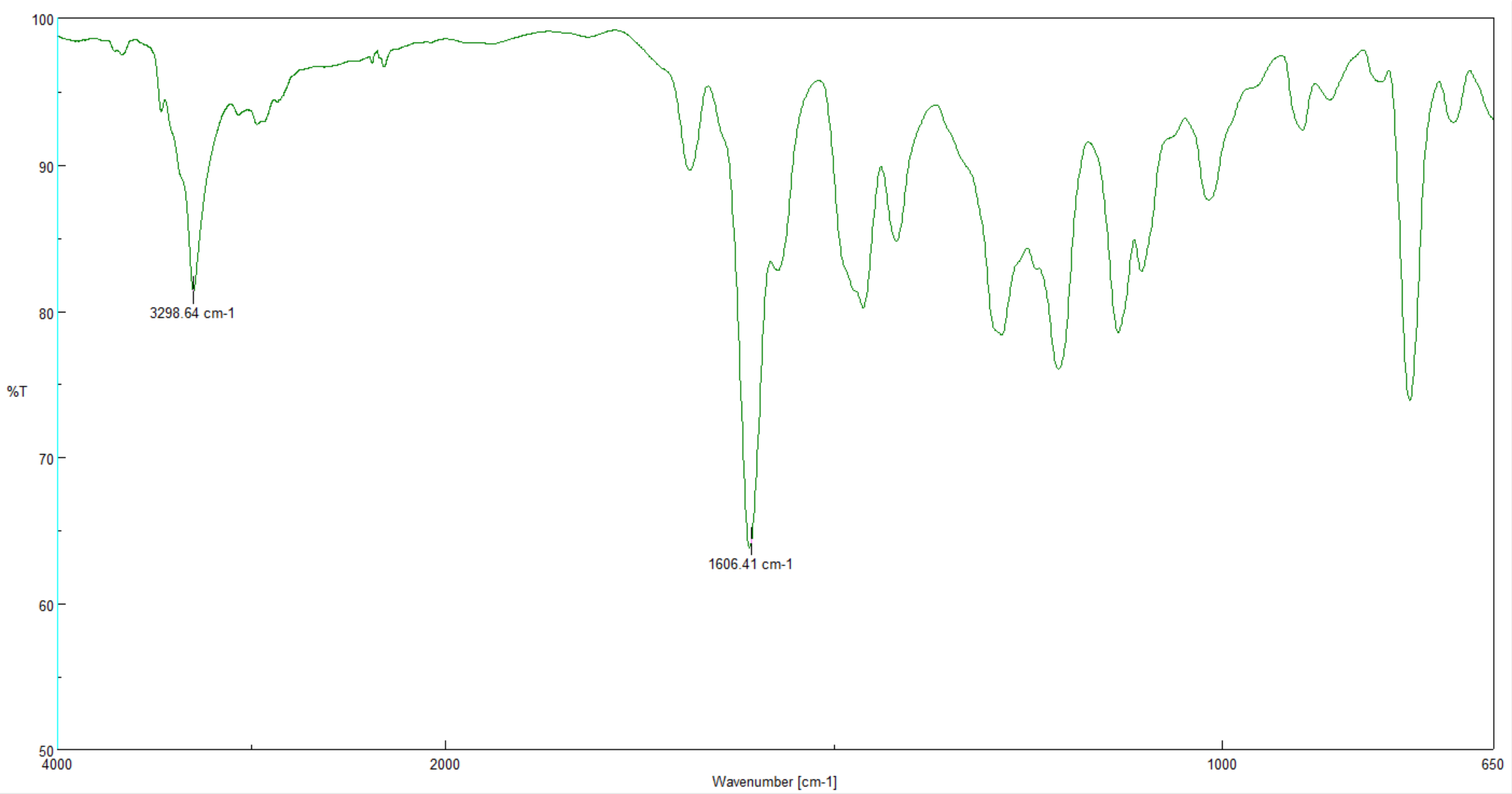

Derivative **2**

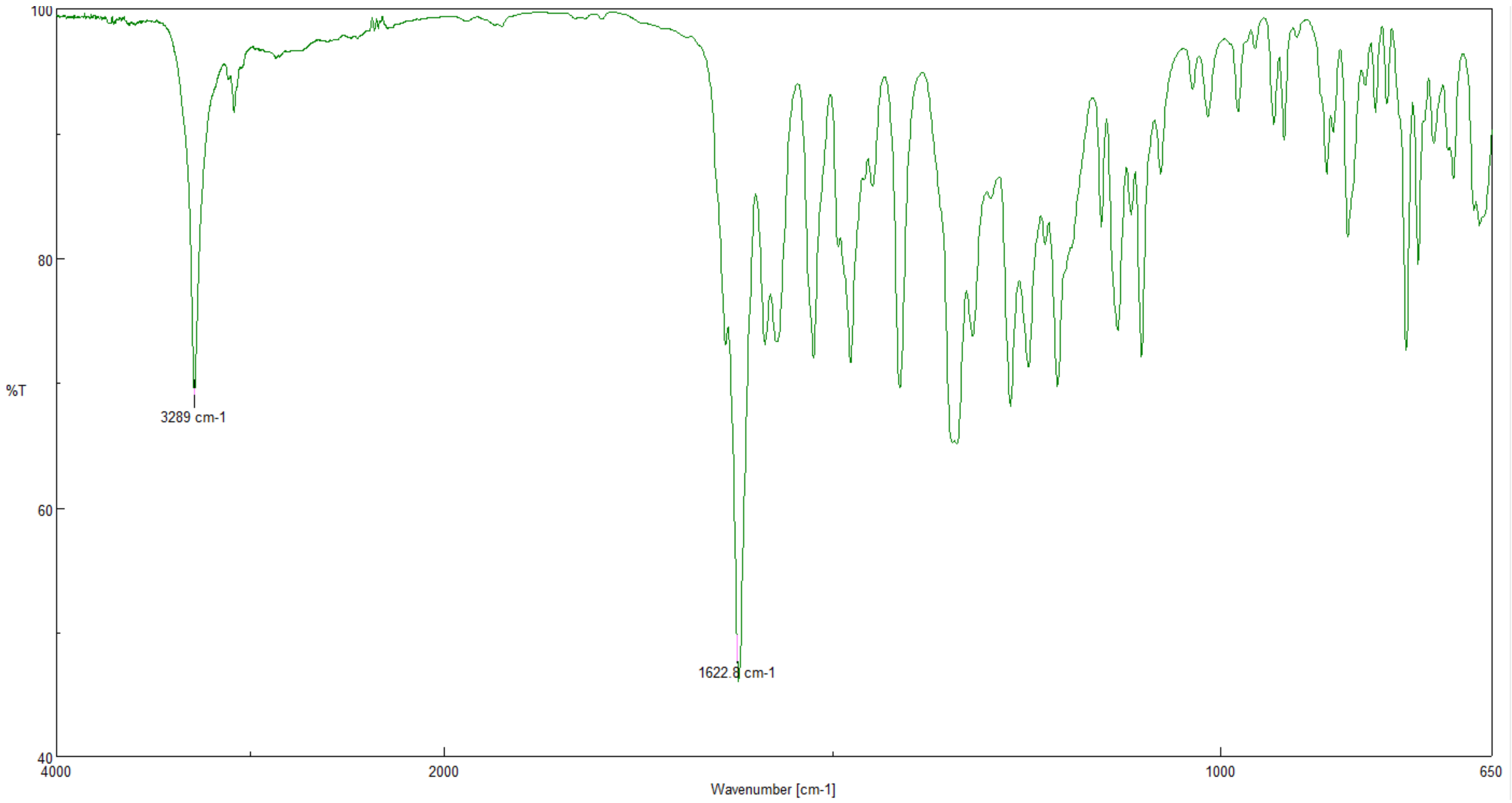

Derivative **3**

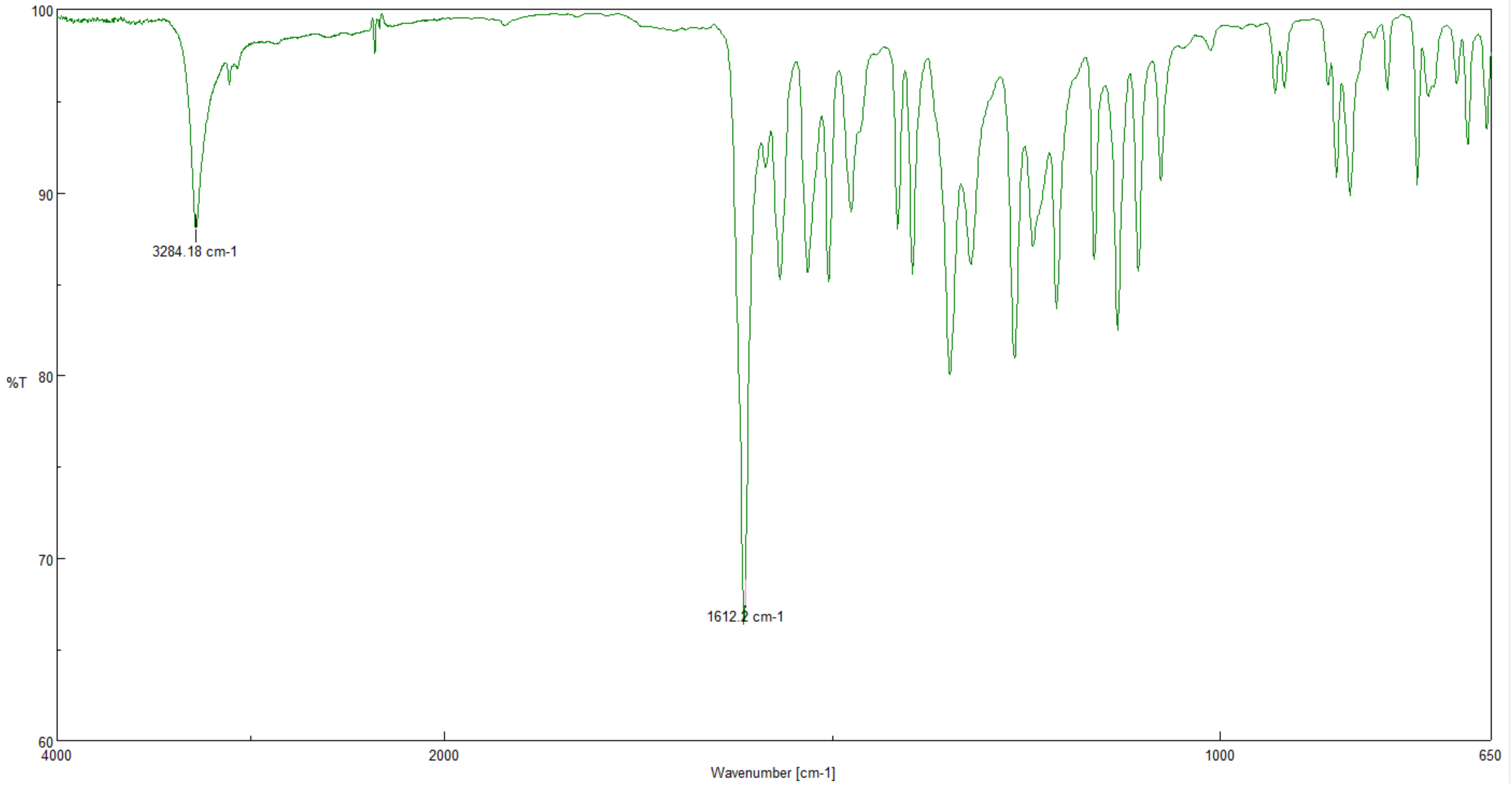

Derivative **4**

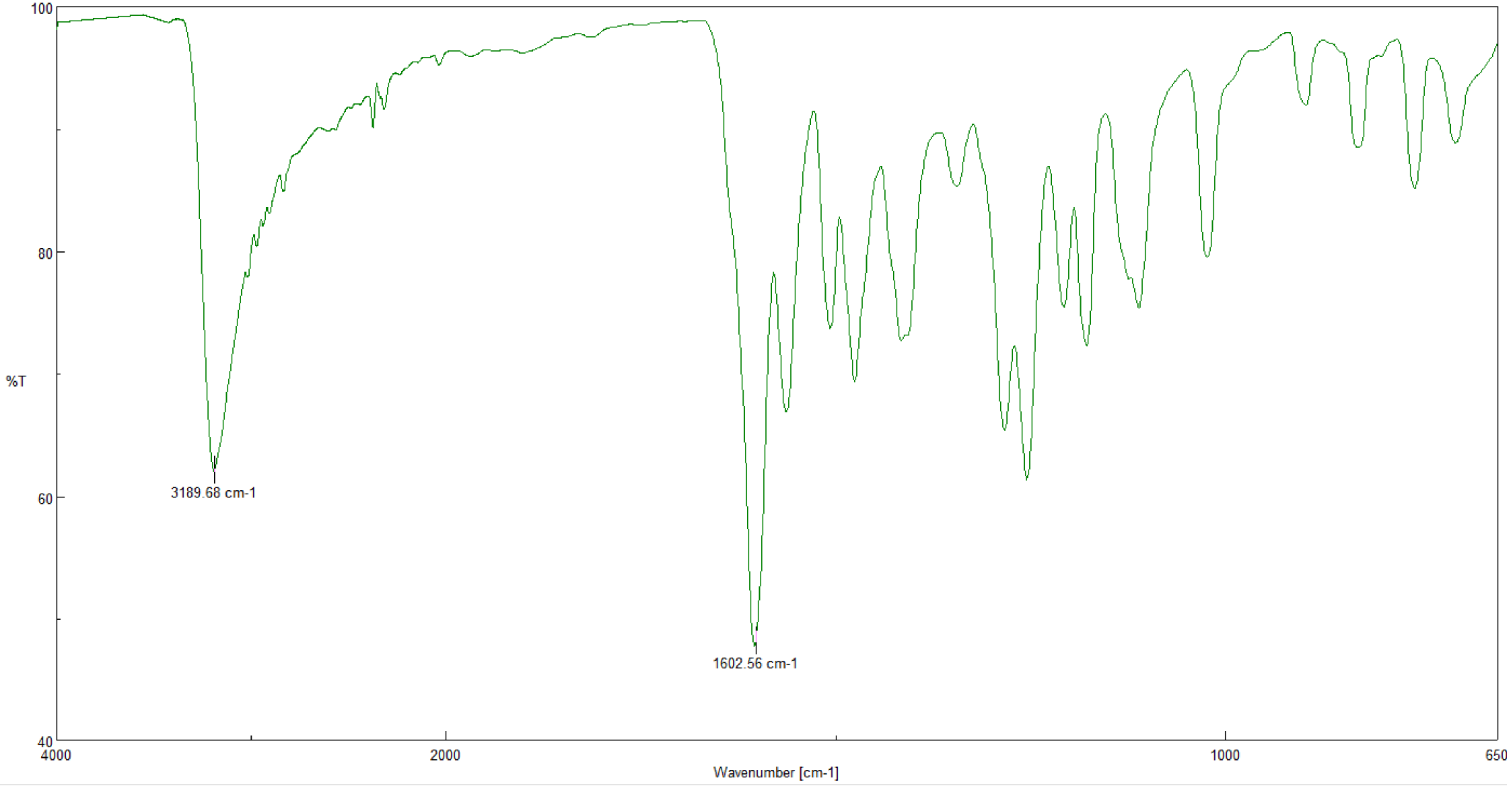

Derivative **5**

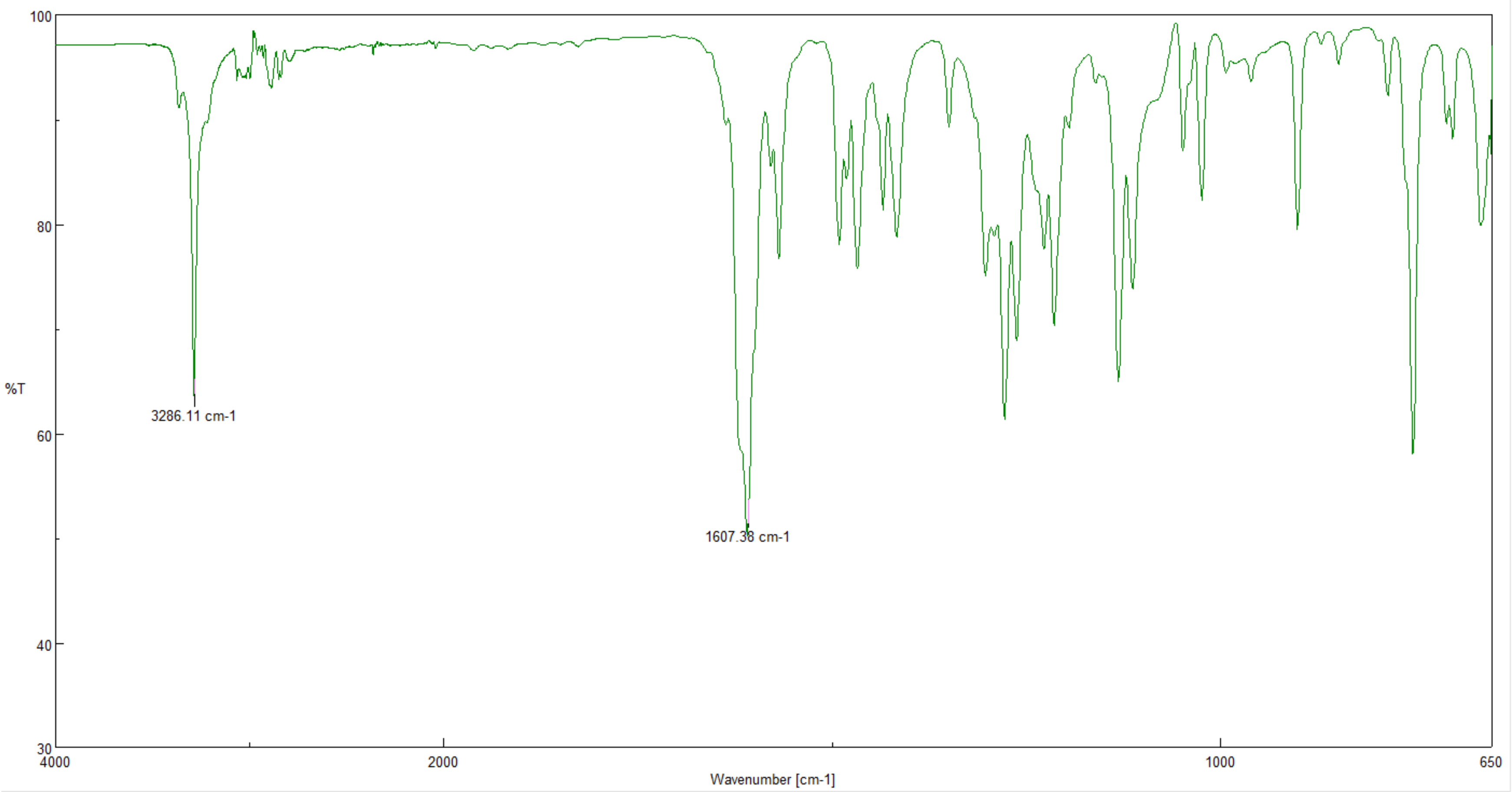

Derivative **6**

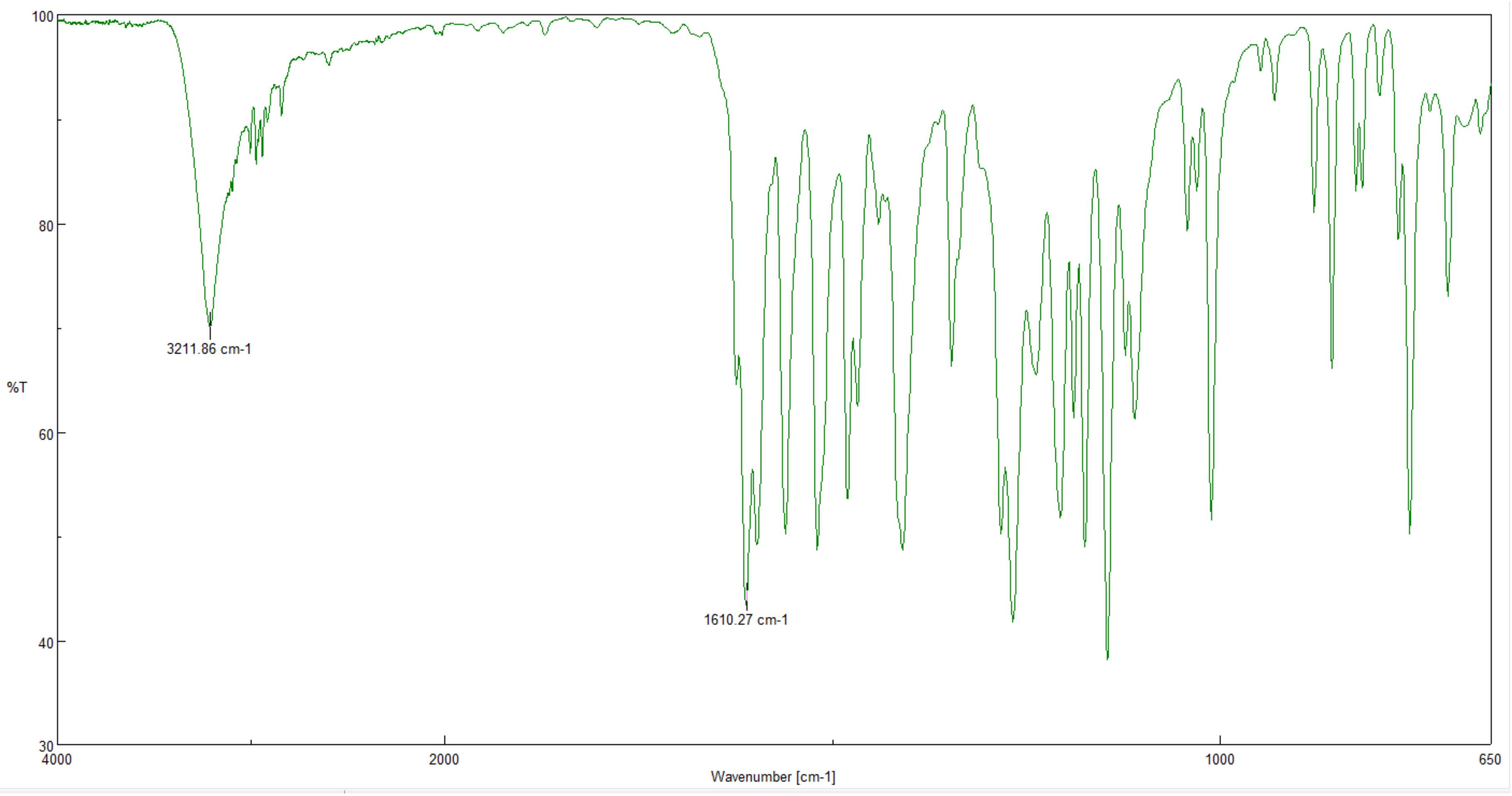

Derivative **7**

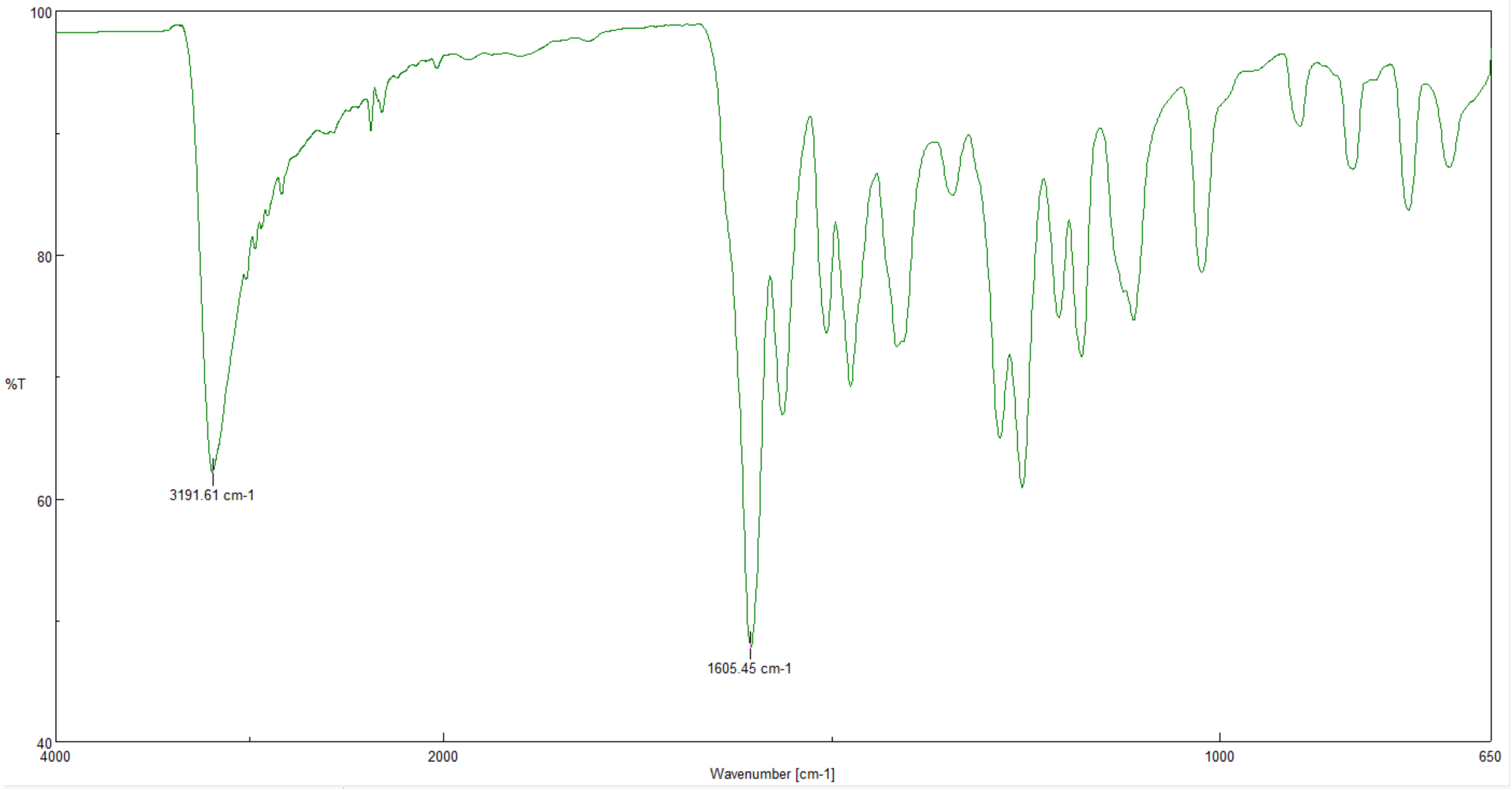

Derivative **8**

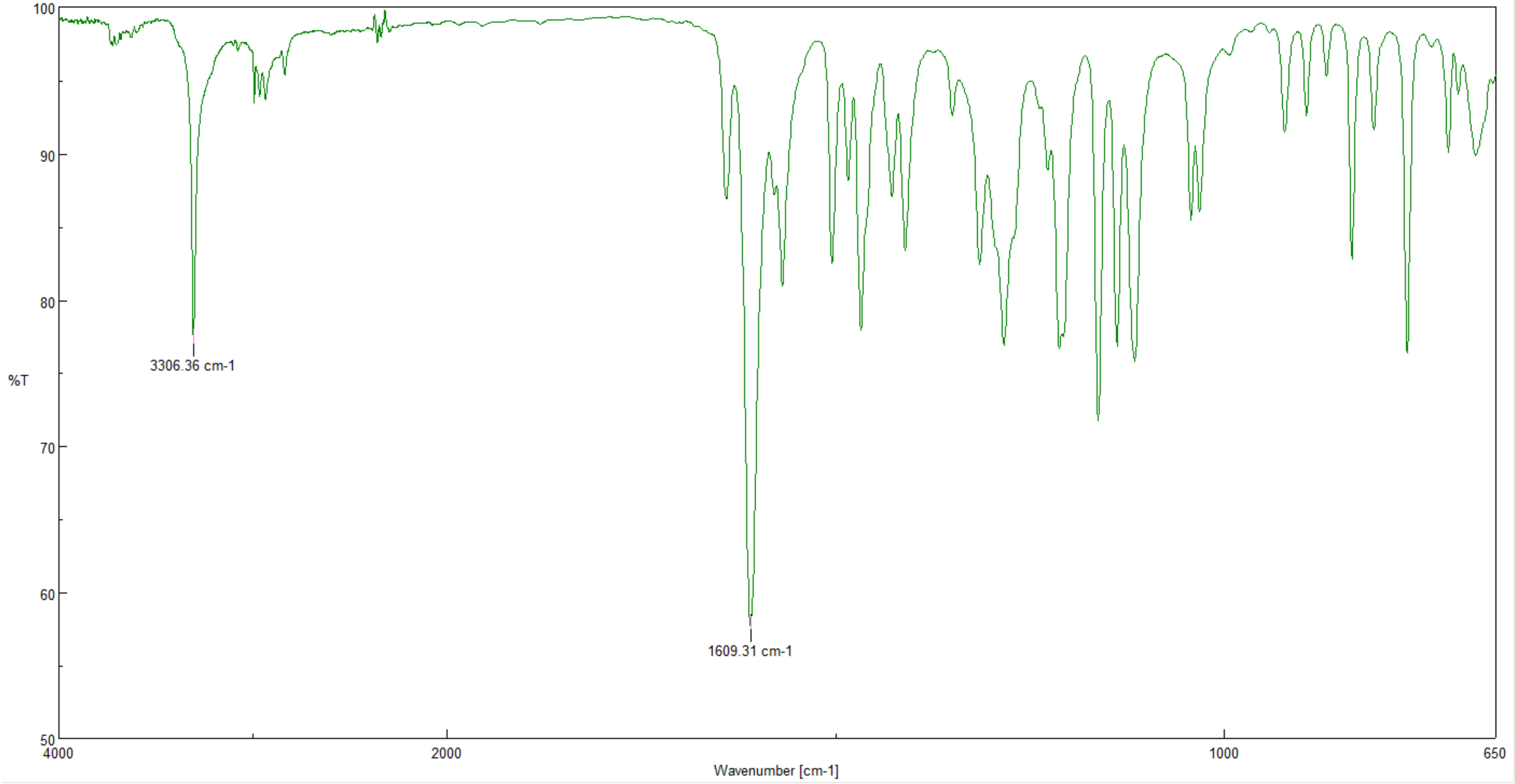

Derivative **9**

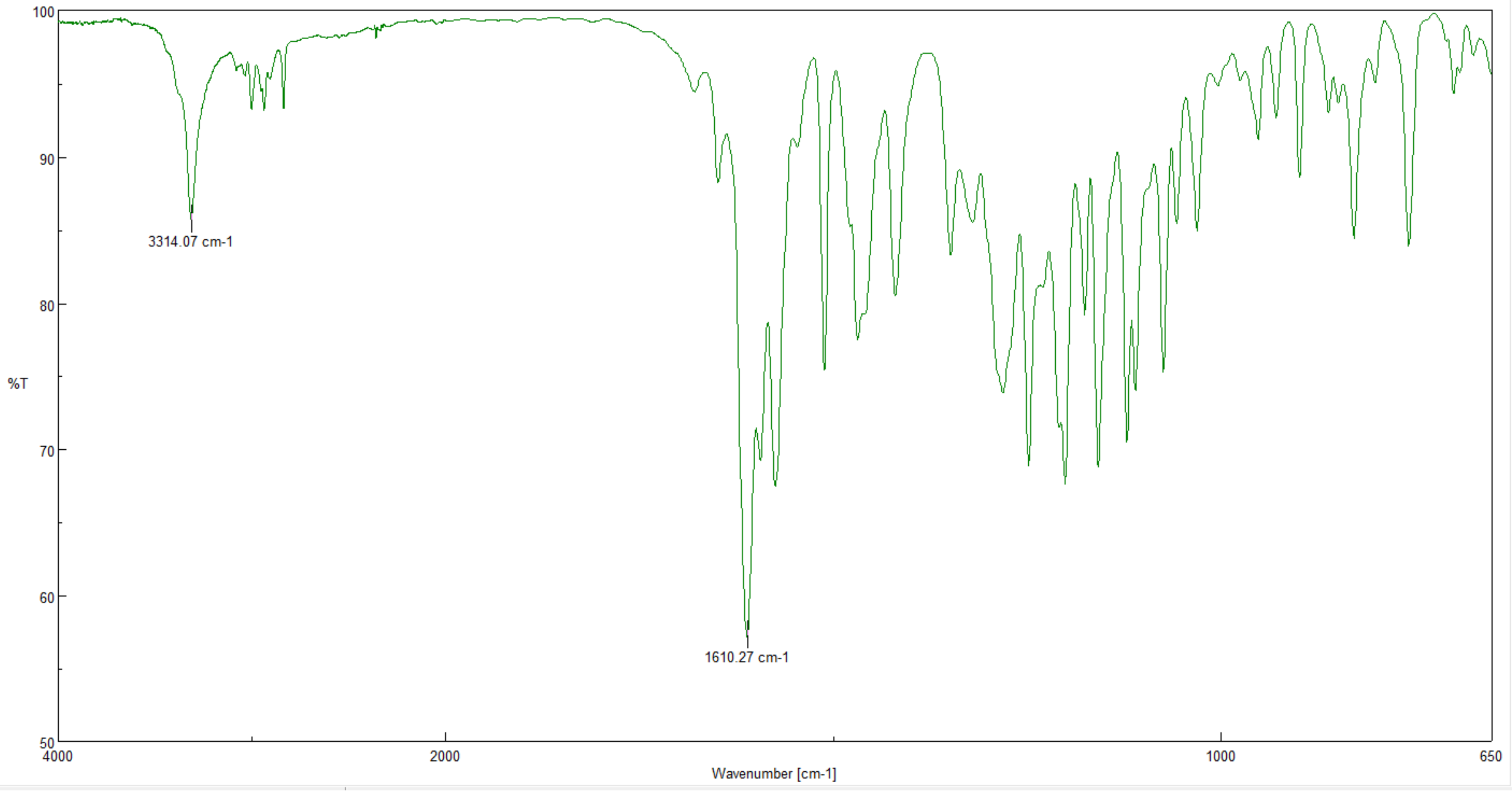

Derivative **10**

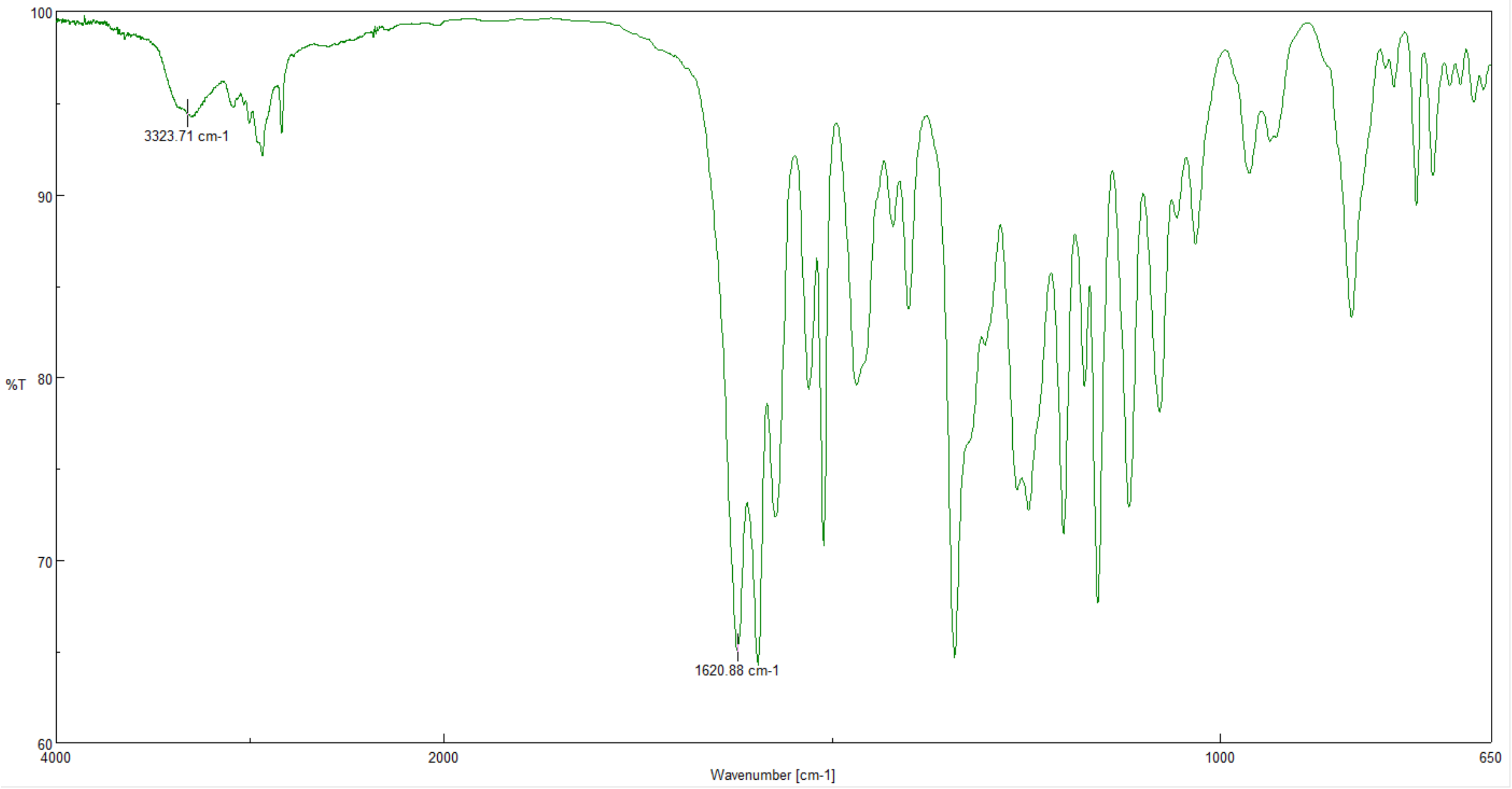

Derivative **11**

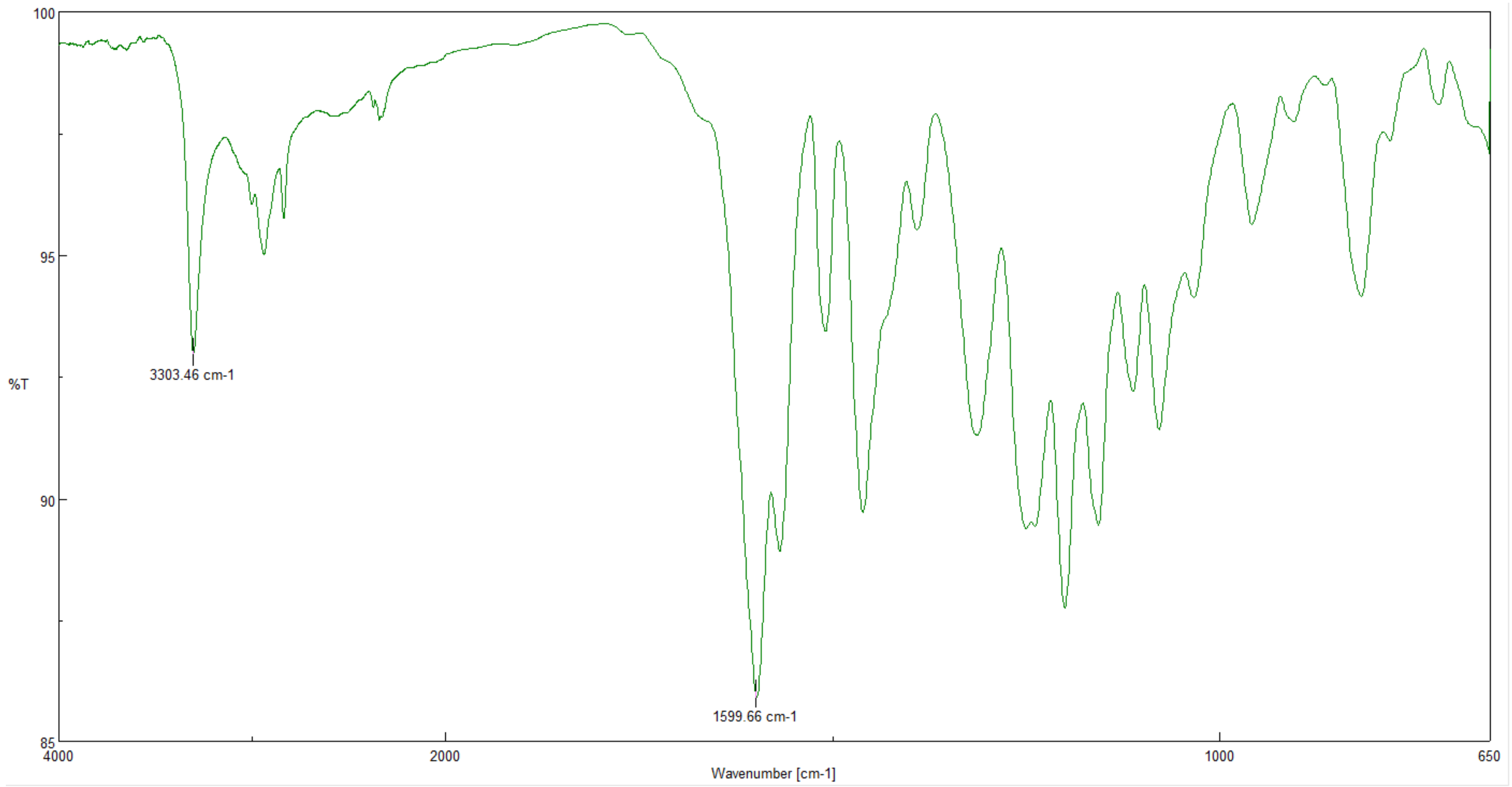

Derivative **12**

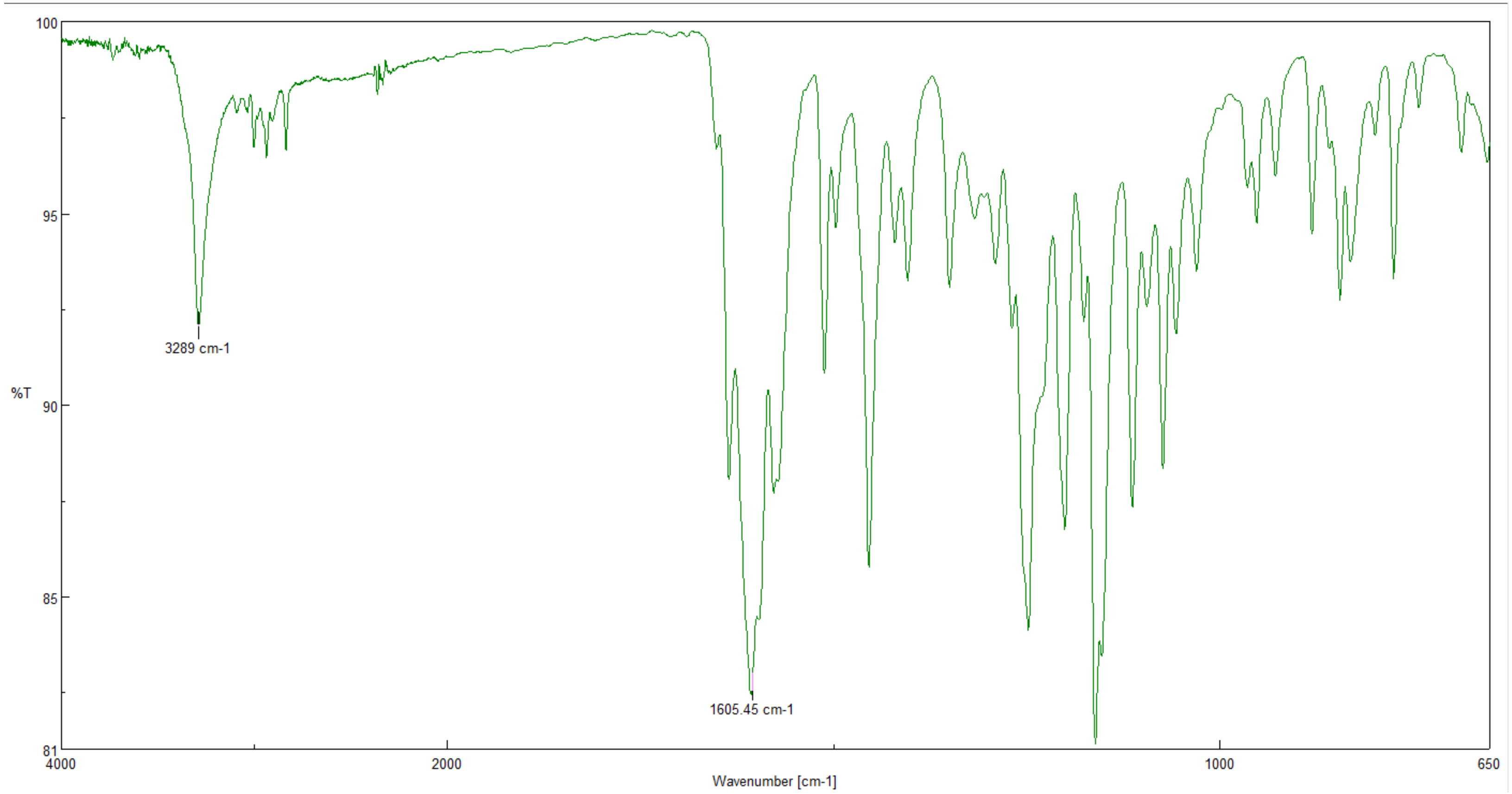

Derivative **13**

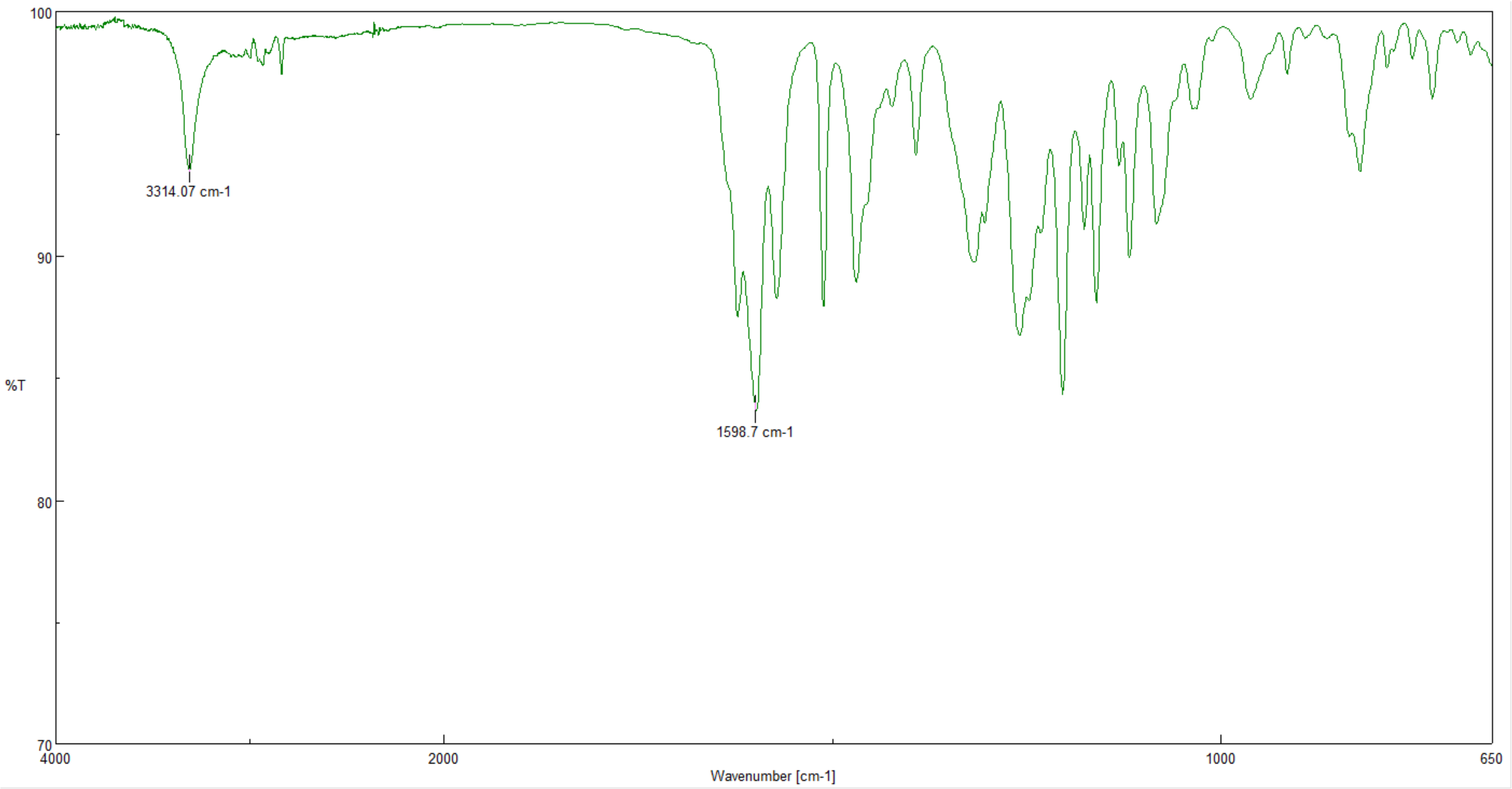

Derivative **14**

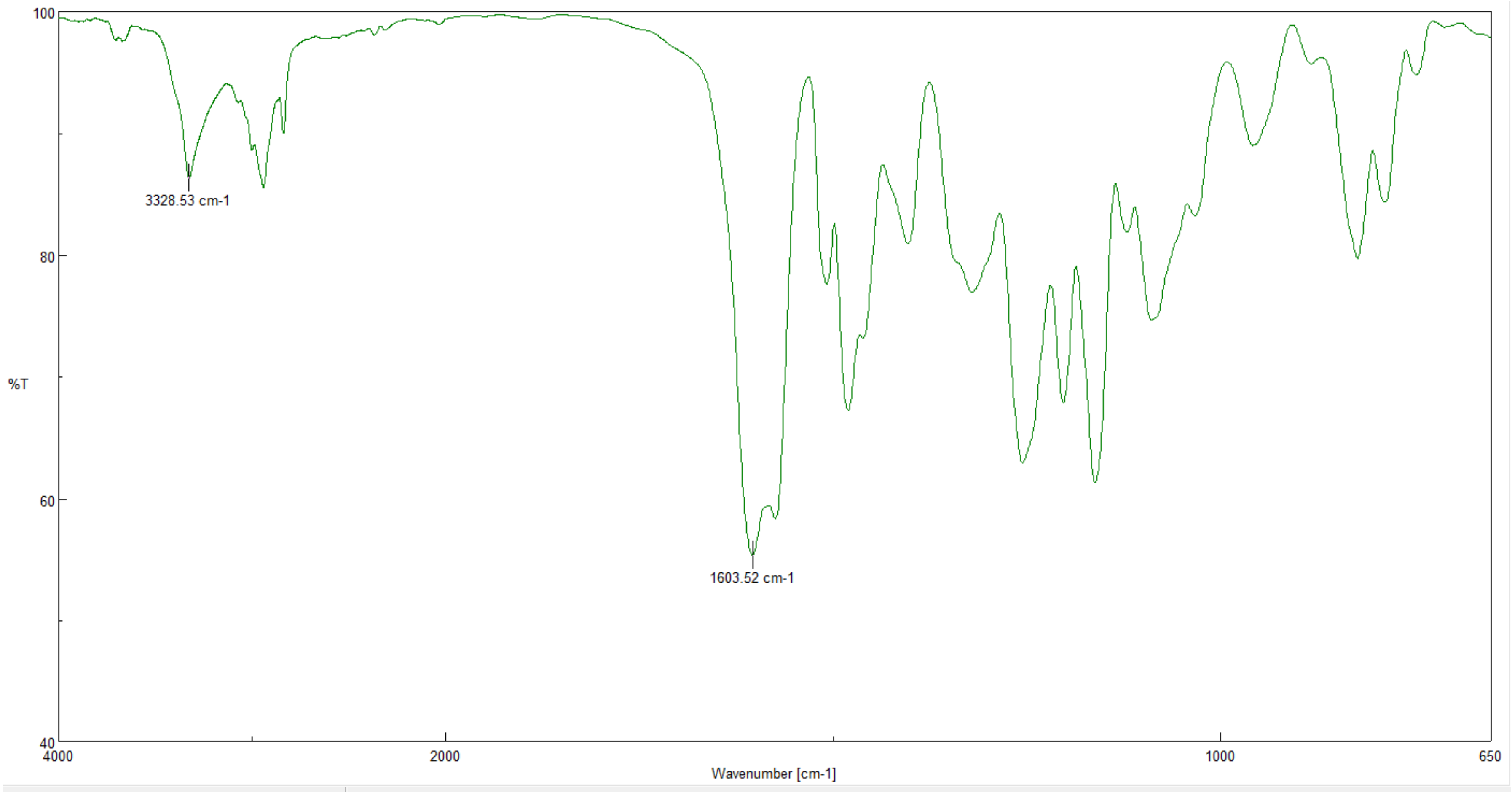

Derivative **15**

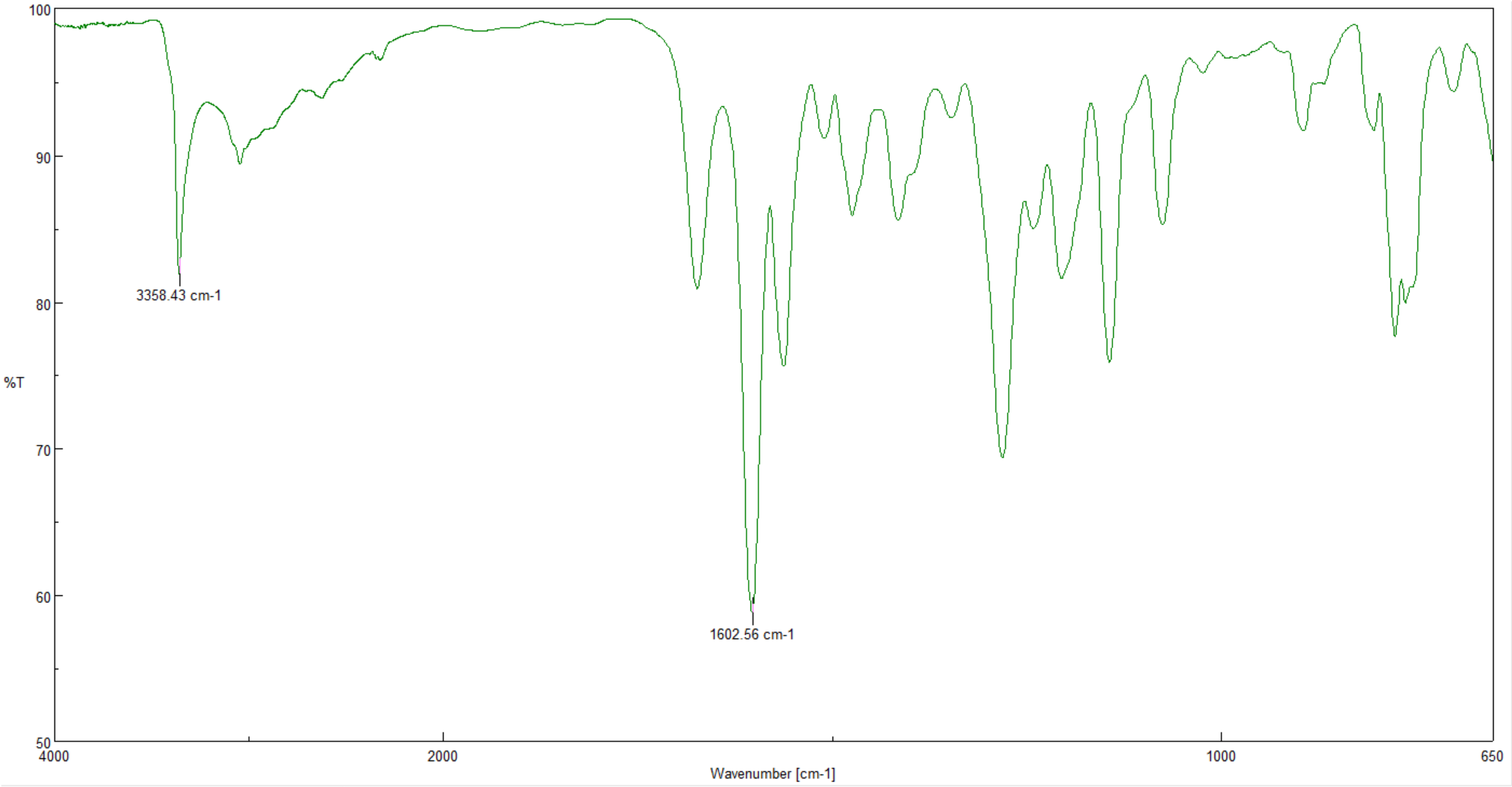

Derivative **16**

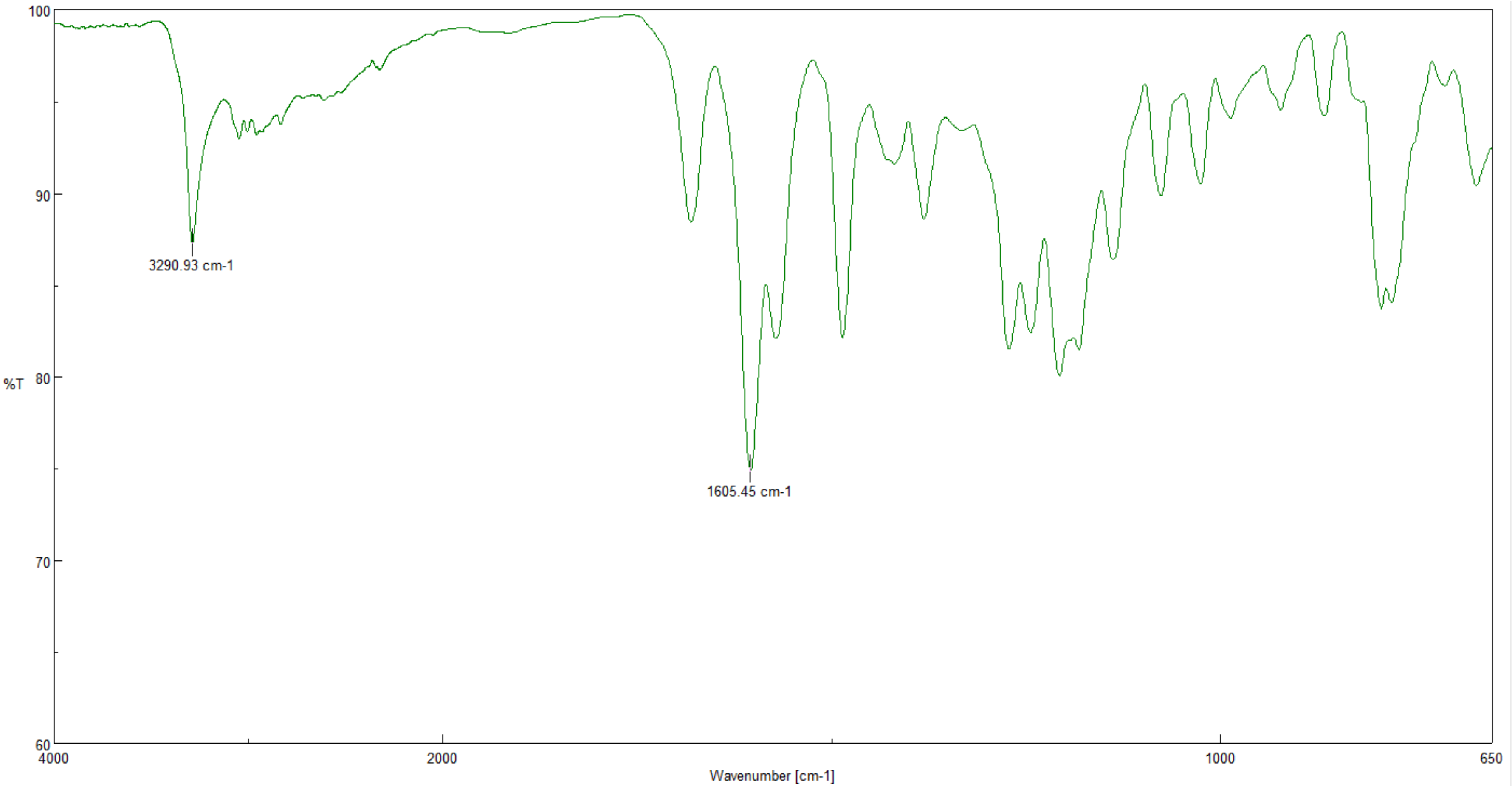

Derivative **17**

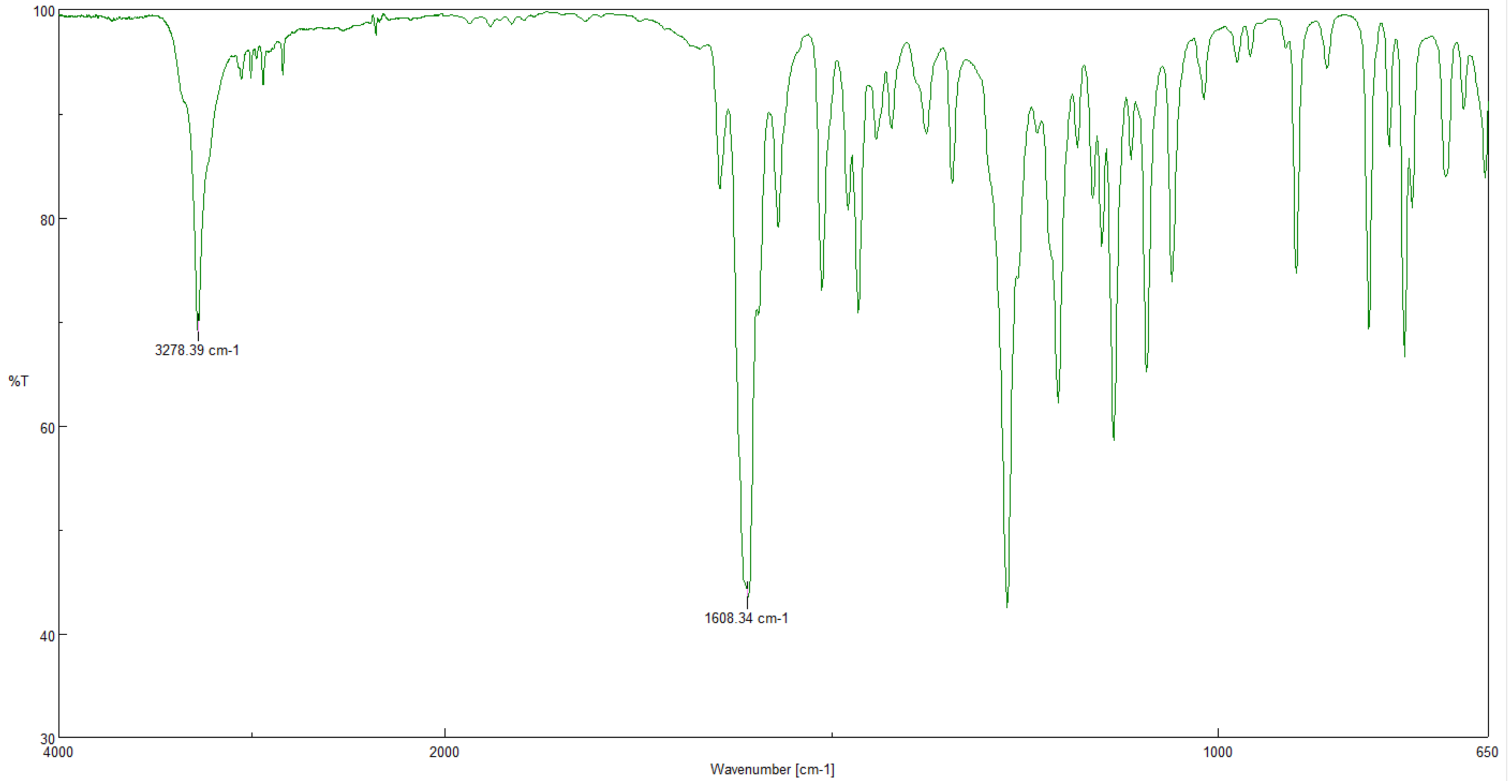

Derivative **18**

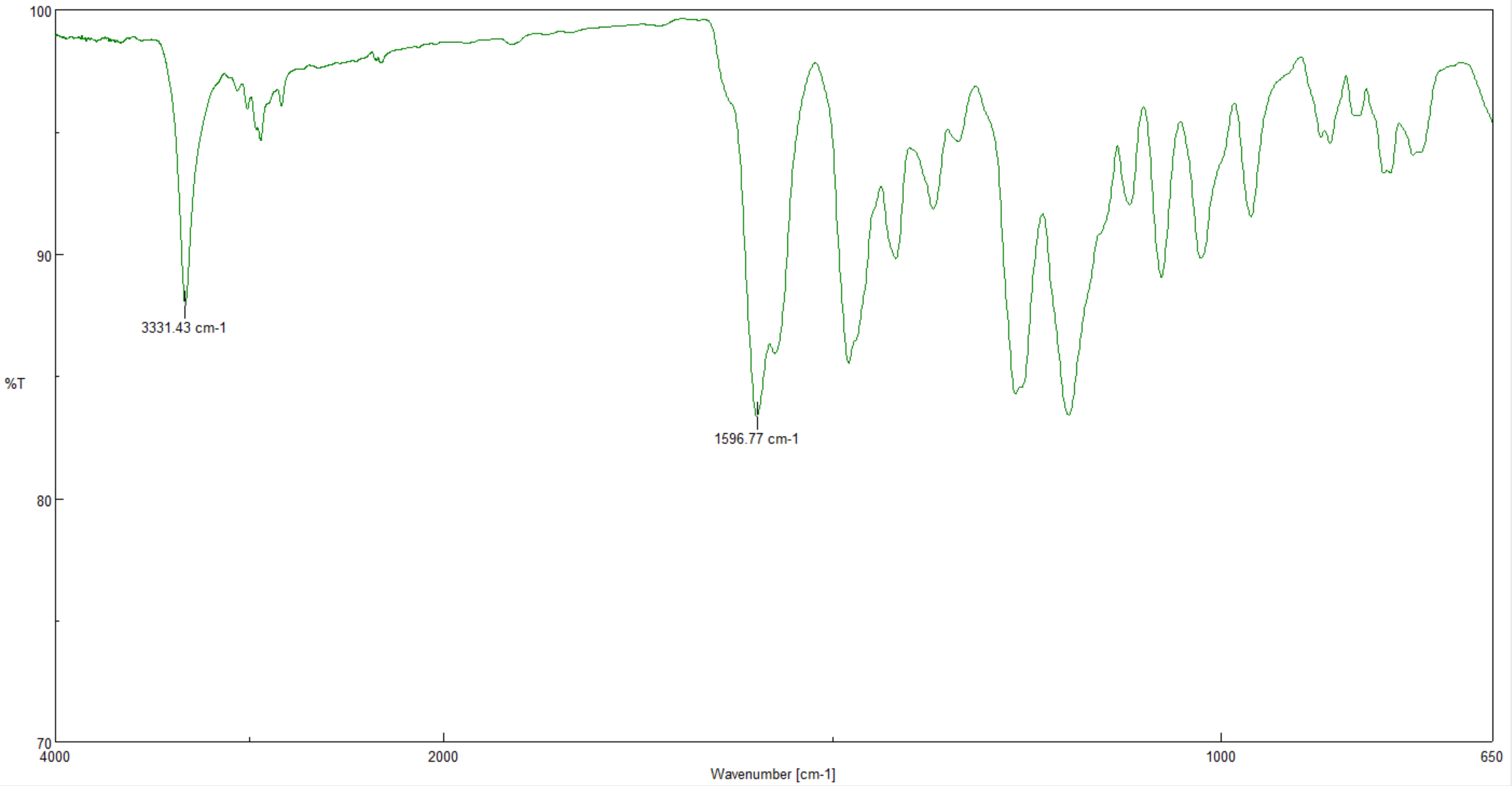

Derivative **19**

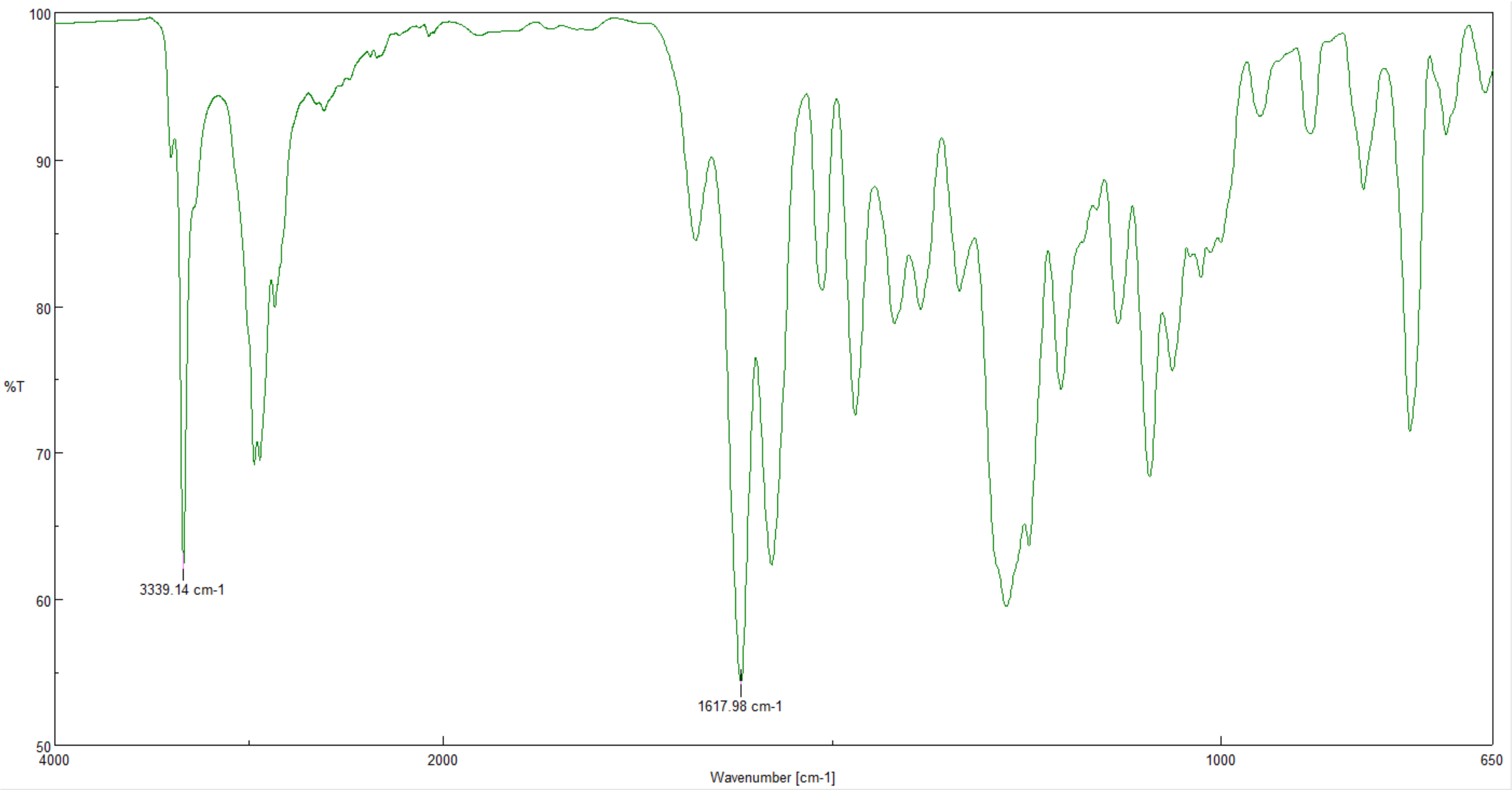

Derivative **20**

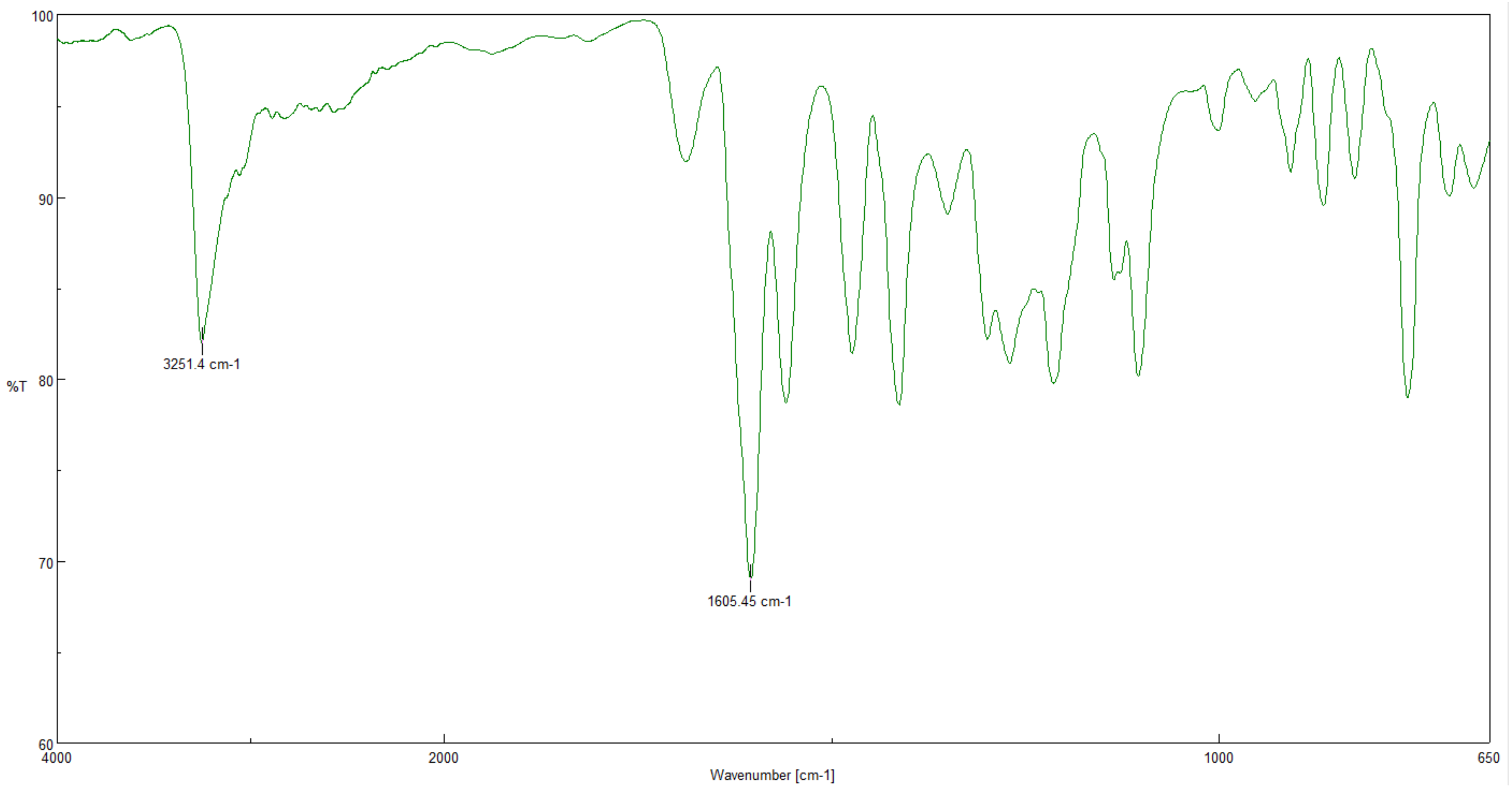

Derivative **21**

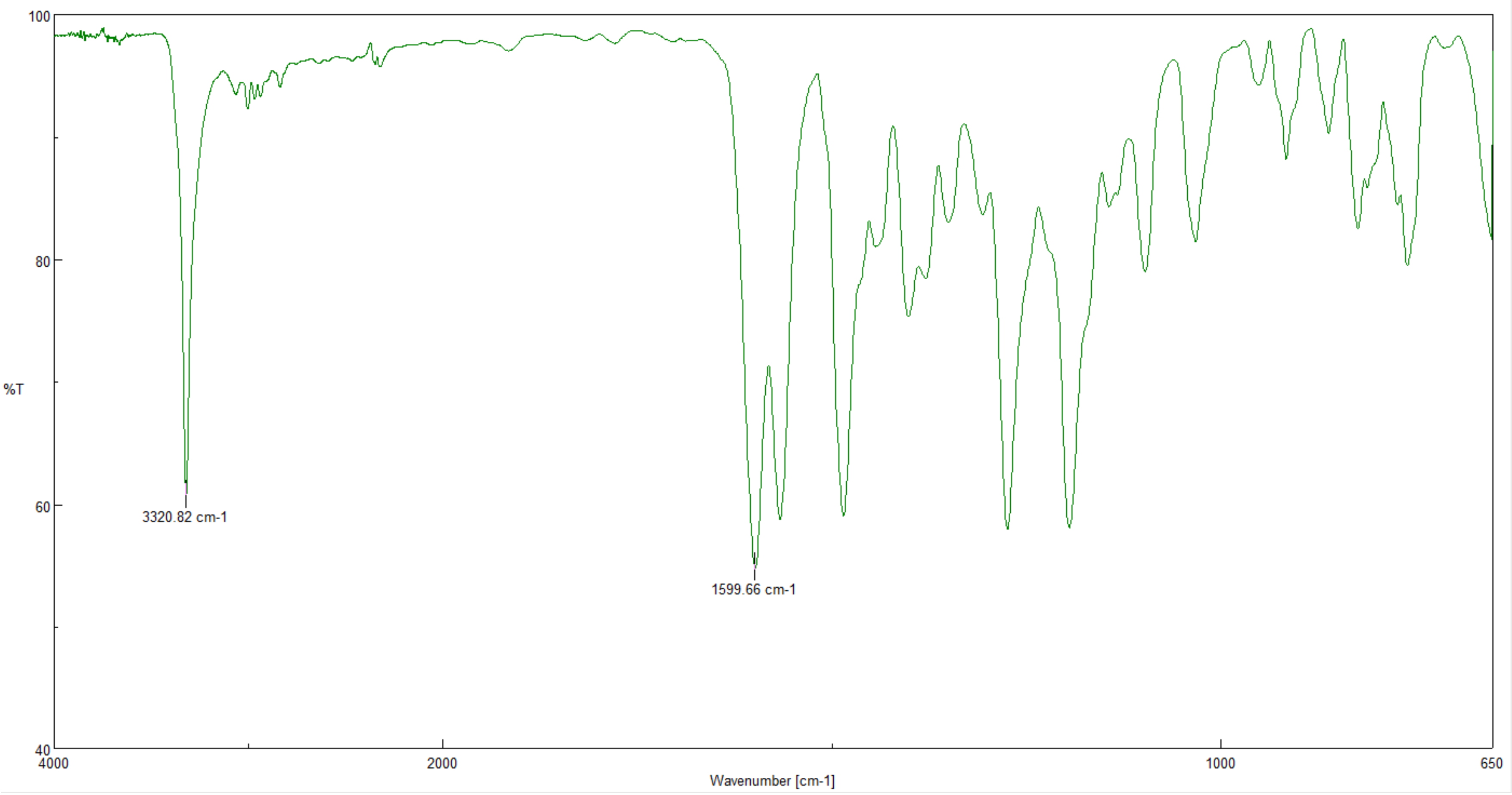

Derivative **22**

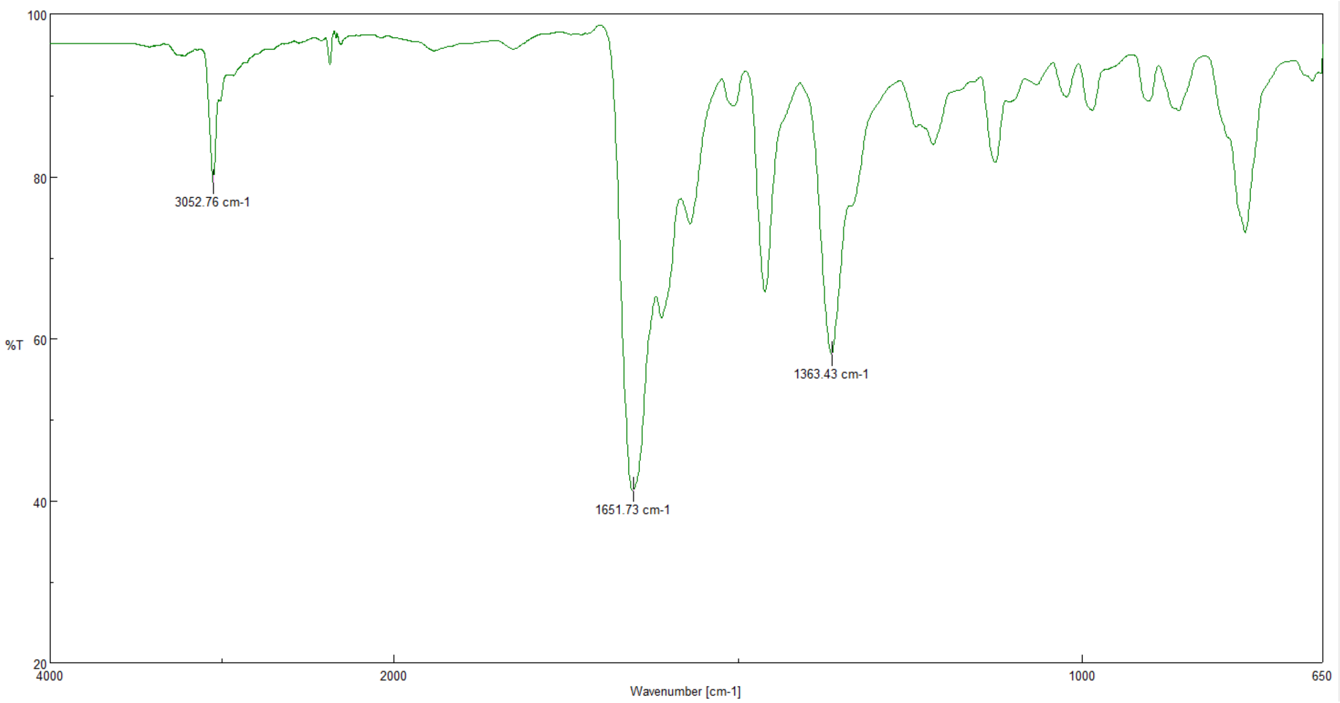

Derivative **23**

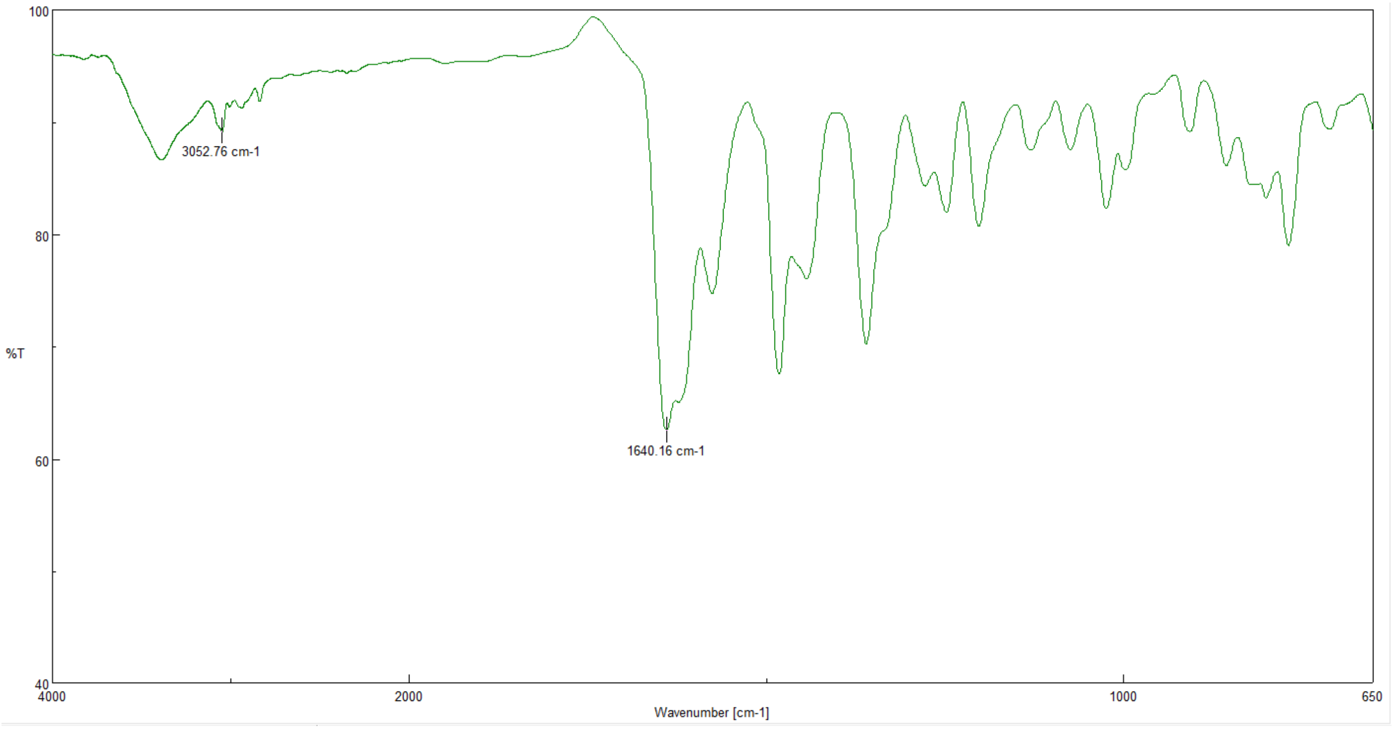

Derivative **24**

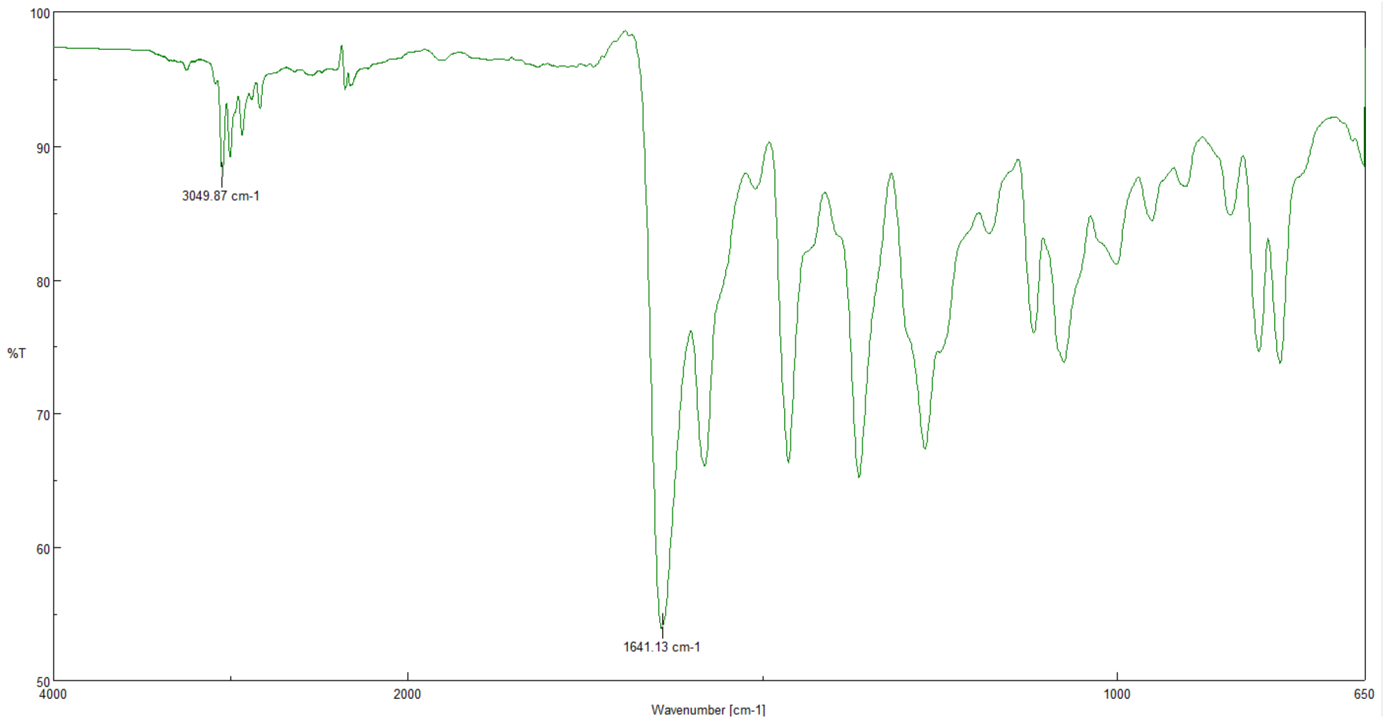

Derivative **25**

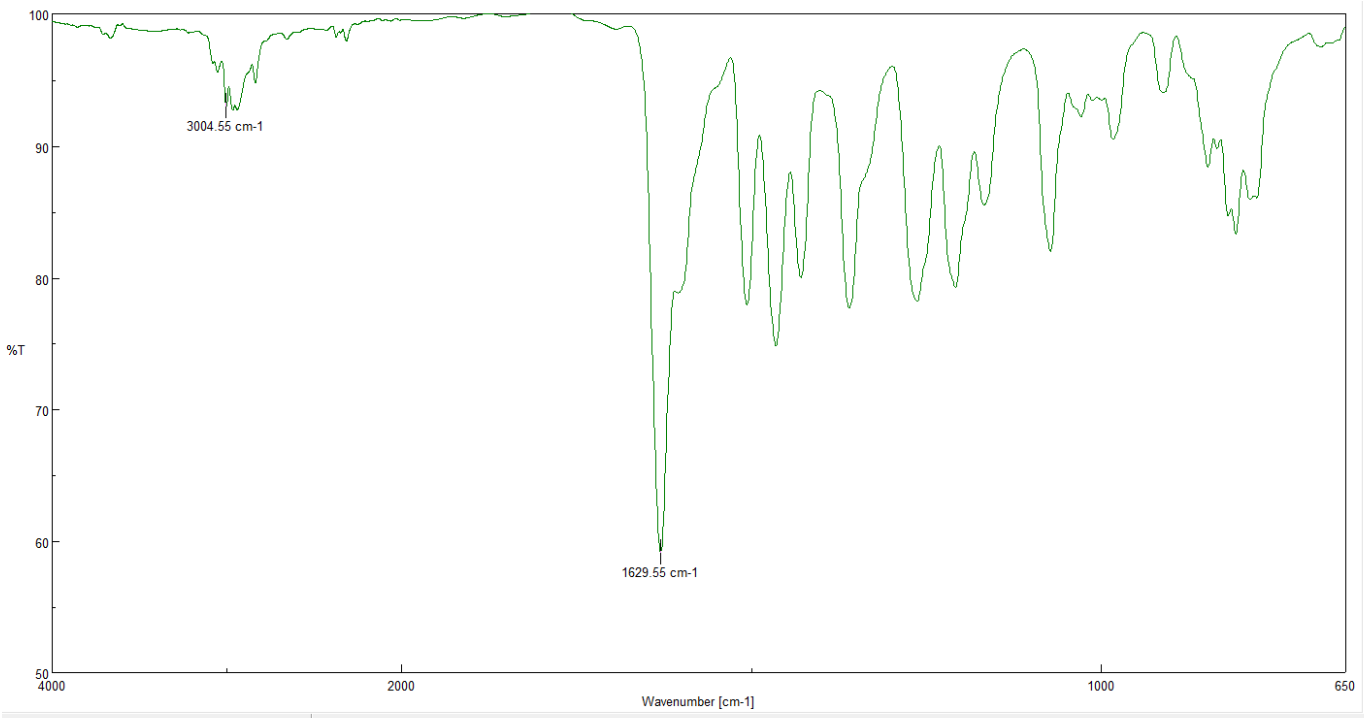

Derivative **26**

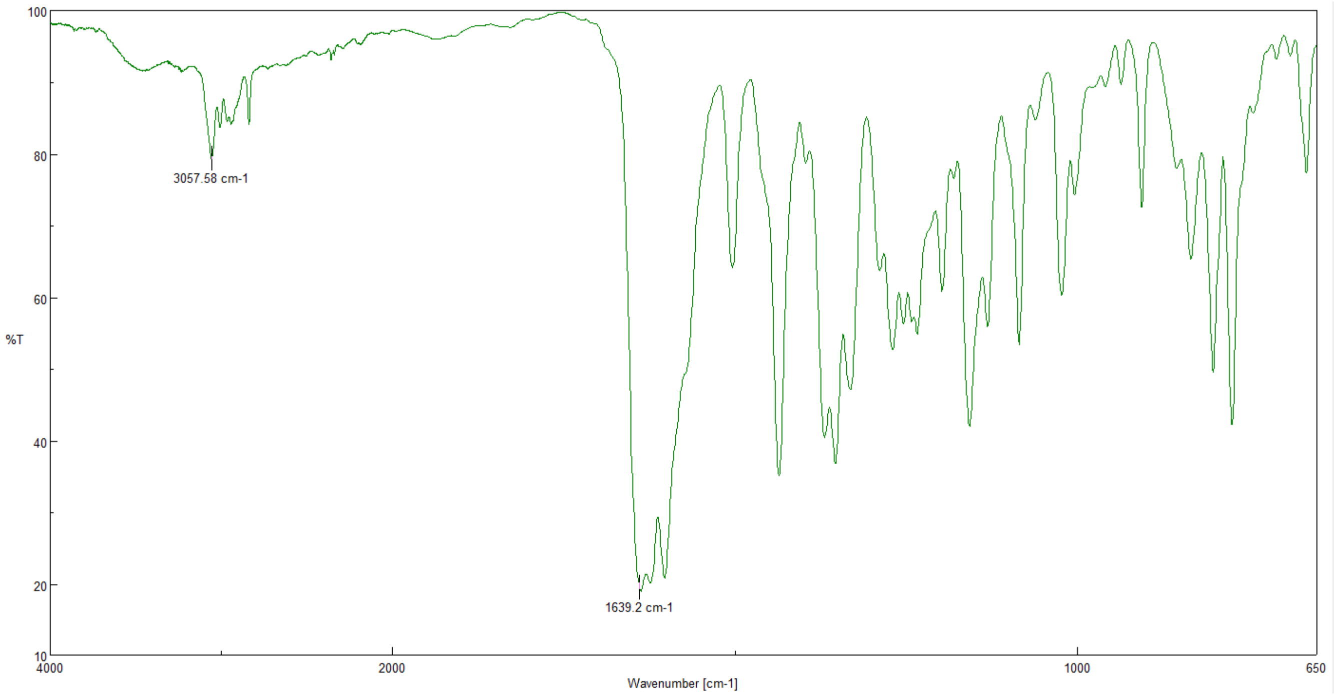

Derivative **27**

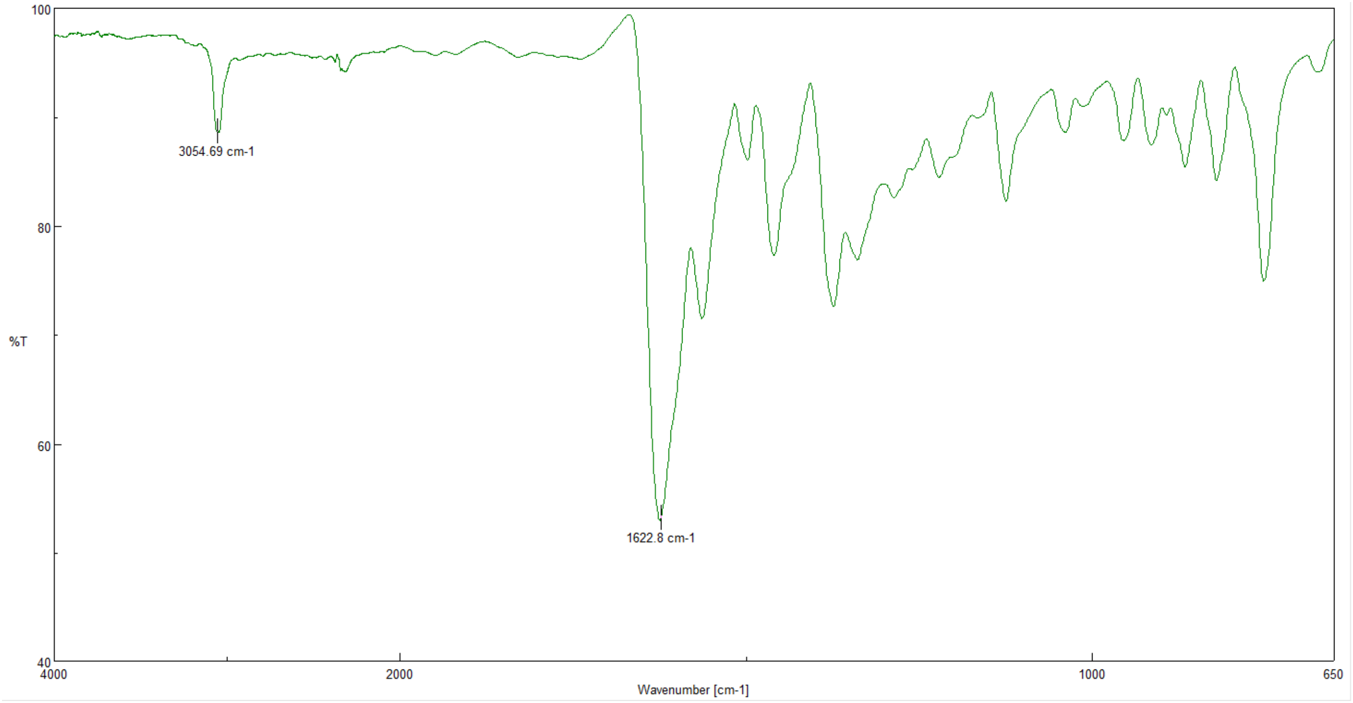

Derivative **28**

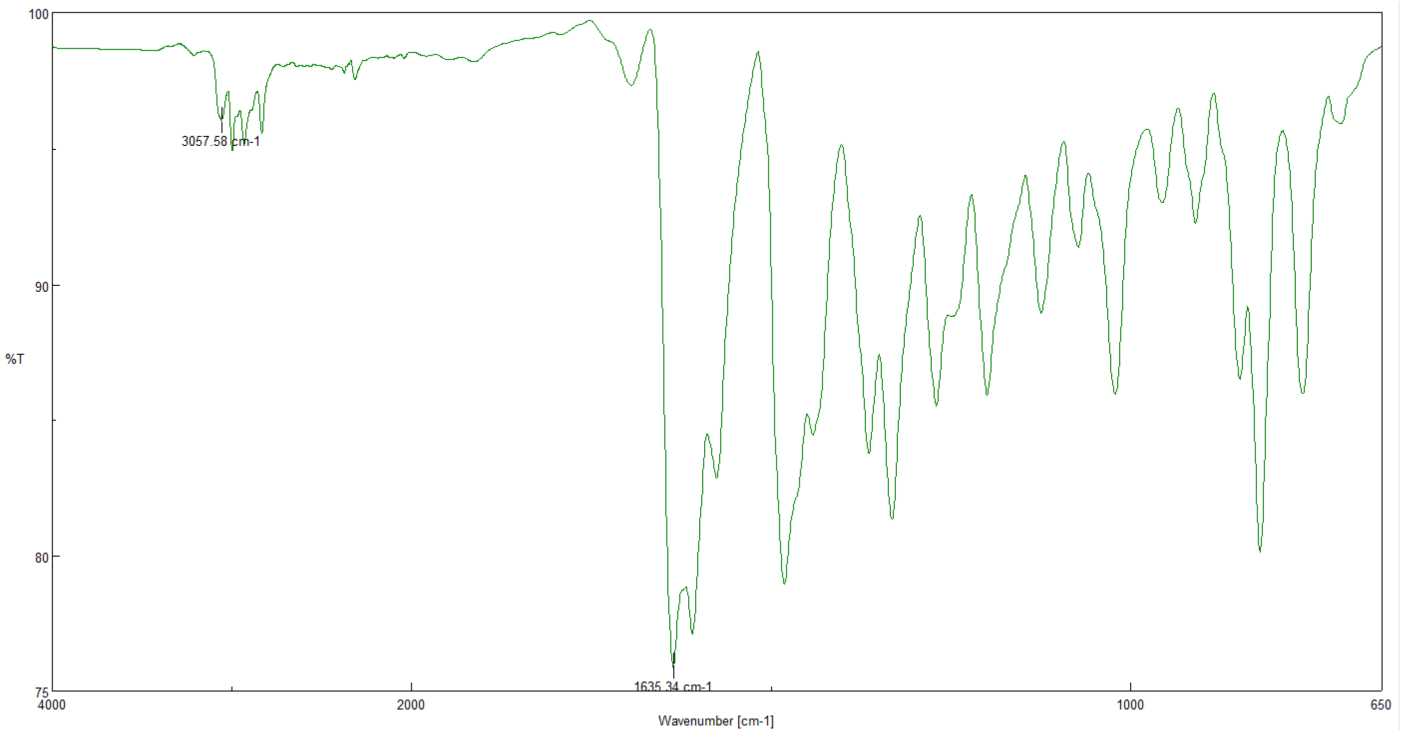

Derivative **29**

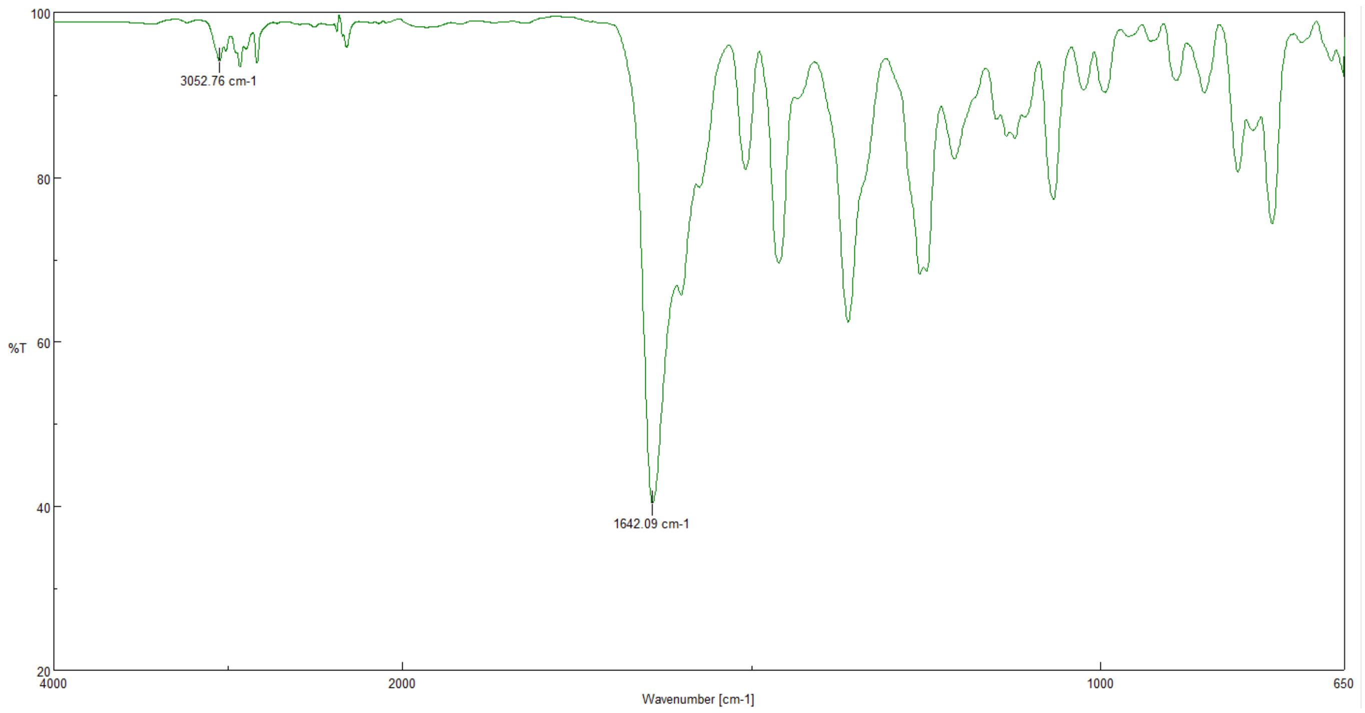

Derivative **30**

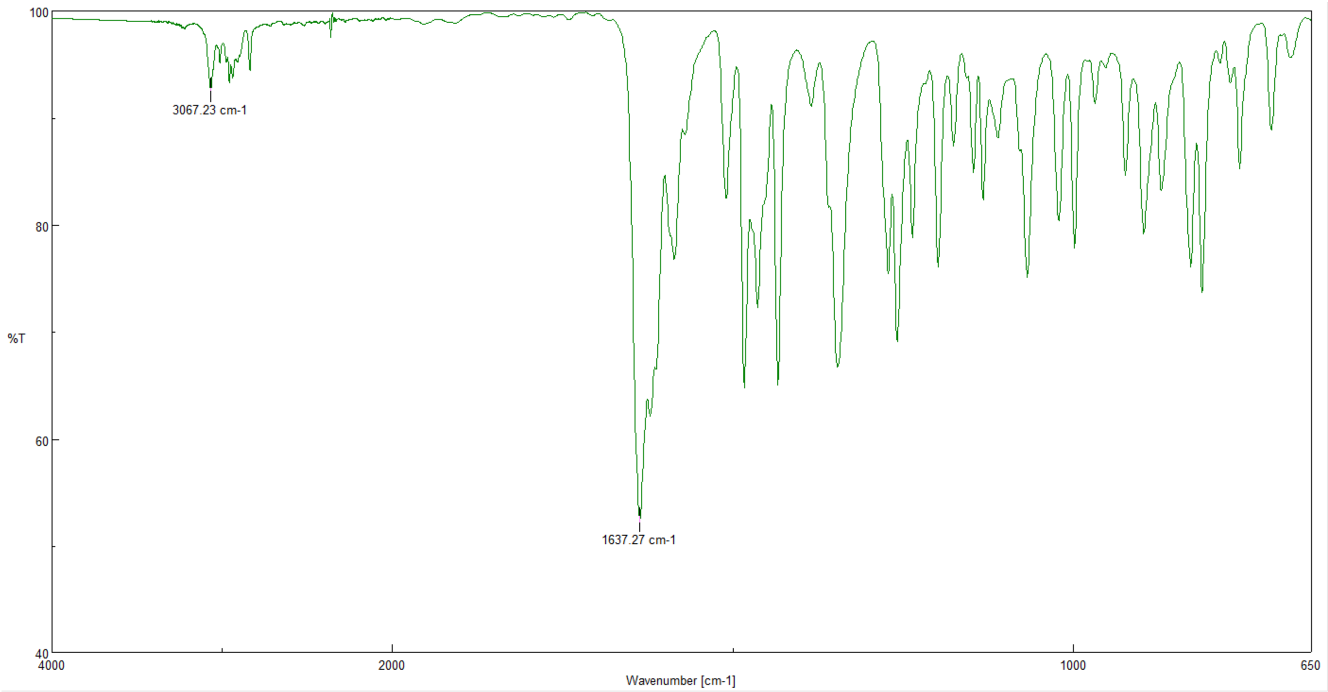

Derivative **31**

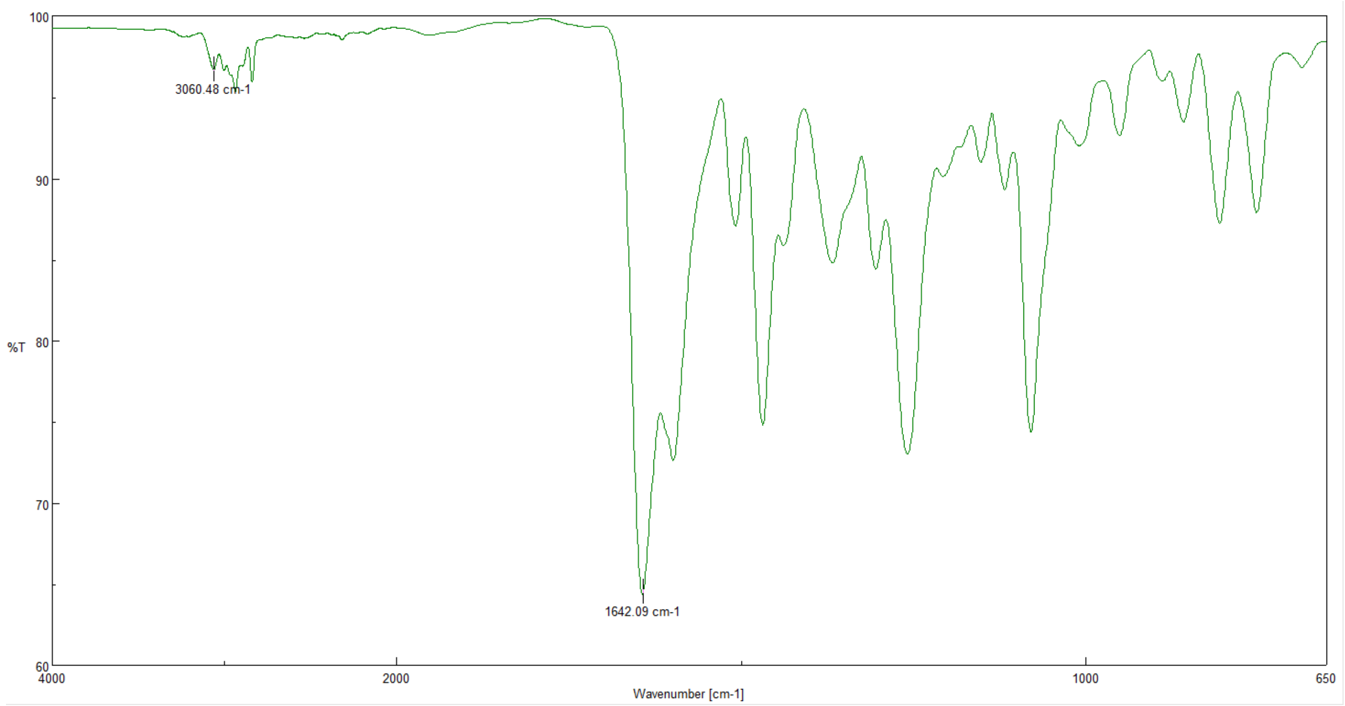

Derivative **32**

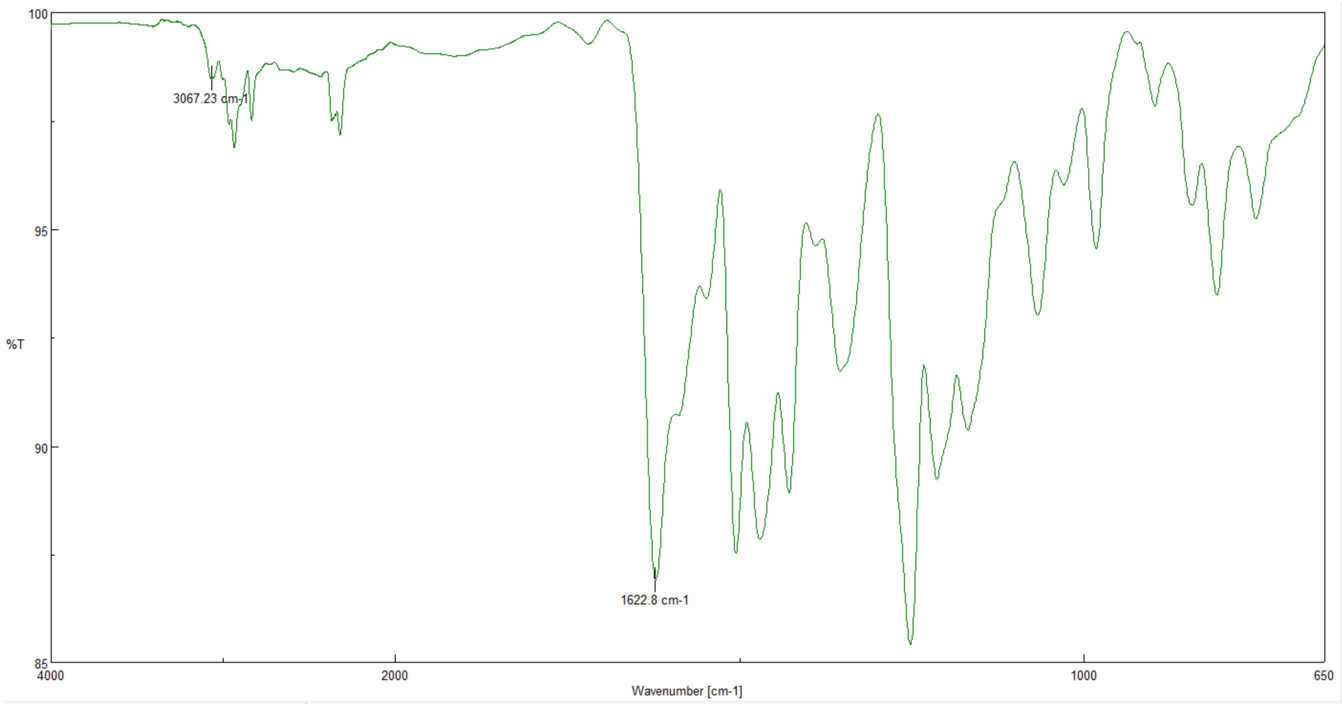

Derivative **33**

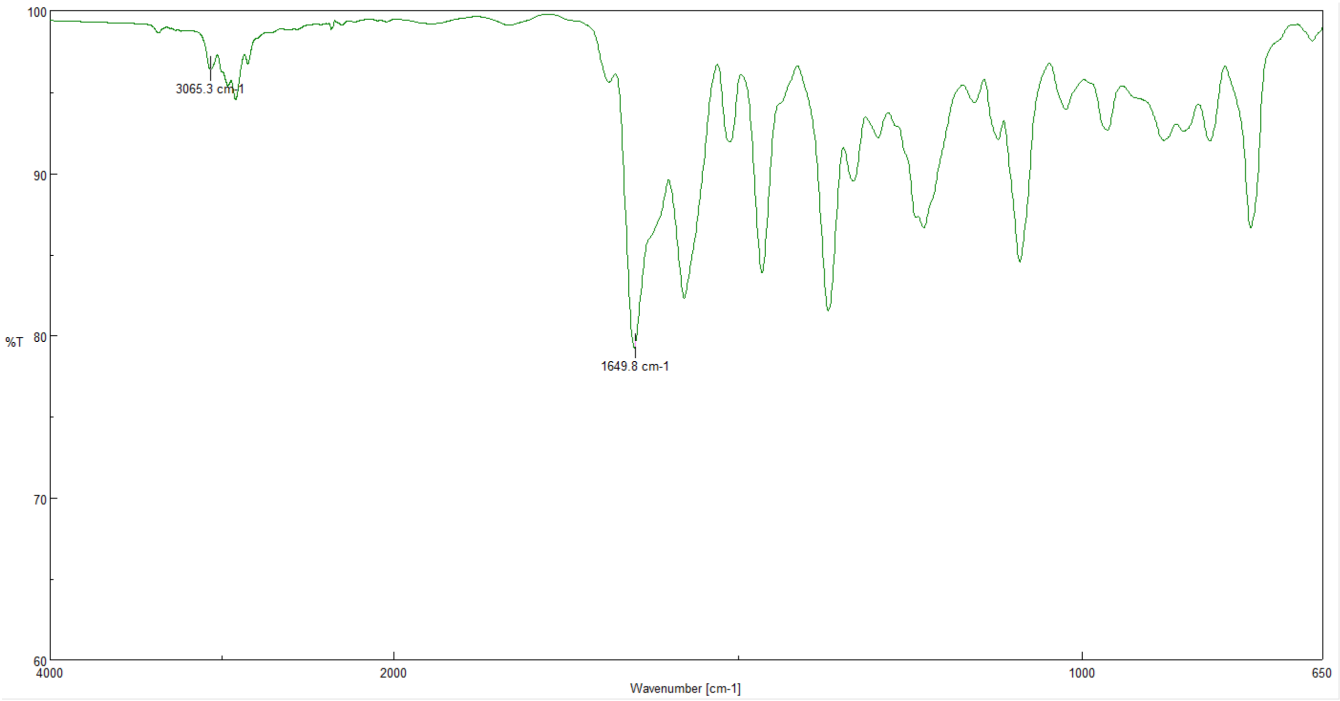

Derivative **34**

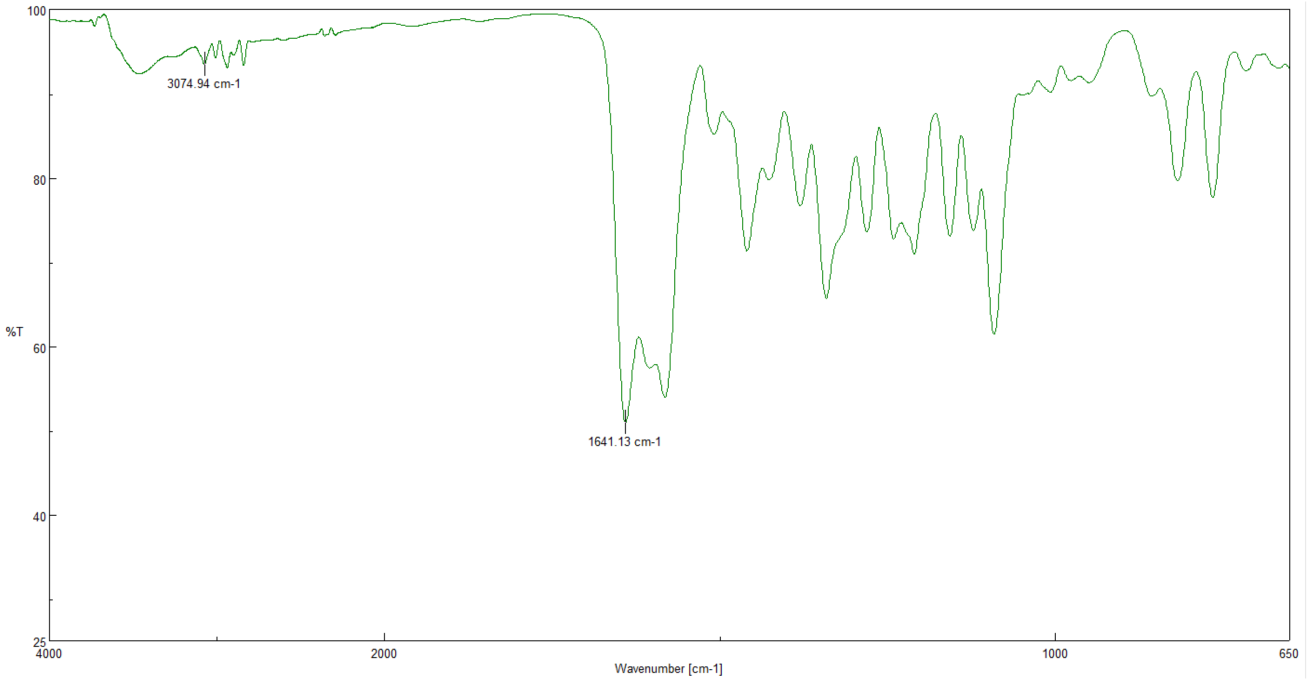

Derivative **35**

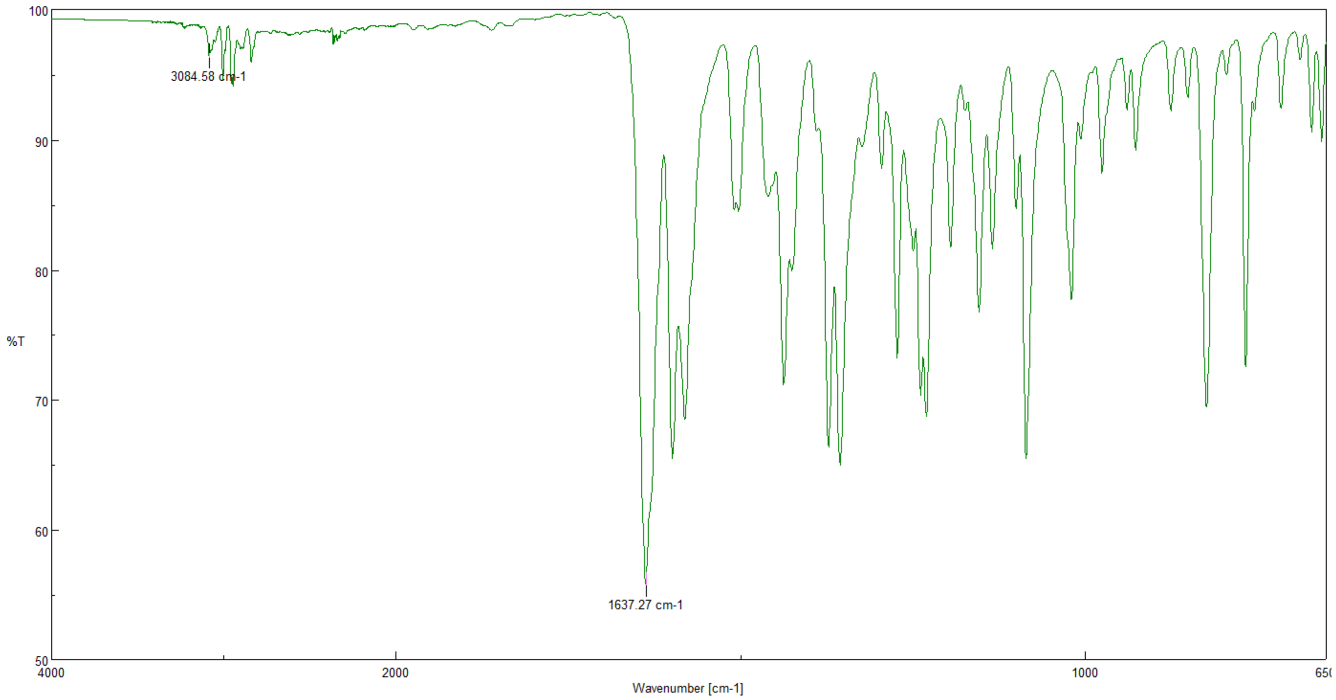

Derivative **36**

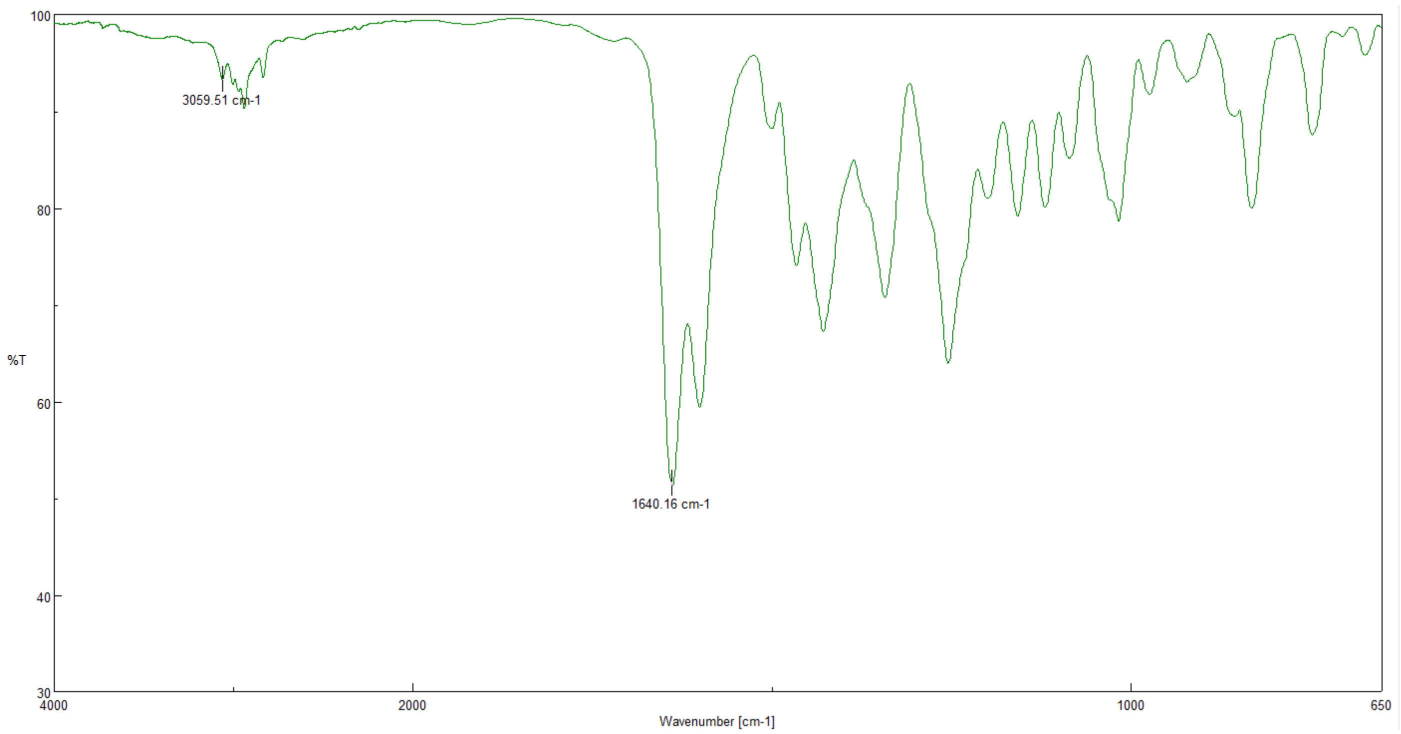

Supplement: Supplementary file 1 [file ijms-19-04086-s001.zip › IR spectra.pdf]
